# Utilizing an artificial intelligence system to build the digital structural proteome of reef-building corals

**DOI:** 10.1093/gigascience/giac117

**Published:** 2022-11-18

**Authors:** Yunchi Zhu, Xin Liao, Tingyu Han, J-Y Chen, Chunpeng He, Zuhong Lu

**Affiliations:** State Key Laboratory of Bioelectronics, Southeast University, Nanjing, Jiangsu, 210096, China; Guangxi Key Lab of Mangrove Conservation and Utilization, Guangxi Mangrove Research Center, Guangxi Academy of Sciences, Beihai 536000, China; State Key Laboratory of Bioelectronics, Southeast University, Nanjing, Jiangsu, 210096, China; Nanjing Institute of Paleontology and Geology, Chinese Academy of Sciences, Nanjing 210008, China; State Key Laboratory of Bioelectronics, Southeast University, Nanjing, Jiangsu, 210096, China; State Key Laboratory of Bioelectronics, Southeast University, Nanjing, Jiangsu, 210096, China

**Keywords:** reef-building coral, ColabFold, structural proteomics

## Abstract

**Background:**

Reef-building corals play an important role in the marine ecosystem, and analyzing their proteomes from a structural perspective will exert positive effects on exploring their biology. Here we integrated mass spectrometry with newly published ColabFold to obtain digital structural proteomes of dominant reef-building corals.

**Results:**

Of the 8,382 homologous proteins in *Acropora muricata, Montipora foliosa*, and *Pocillopora verrucosa* identified, 8,166 received predicted structures after about 4,060 GPU hours of computation. The resulting dataset covers 83.6% of residues with a confident prediction, while 25.9% have very high confidence.

**Conclusions:**

Our work provides insight-worthy predictions for coral research, confirms the reliability of ColabFold in practice, and is expected to be a reference case in the impending high-throughput era of structural proteomics.

## Introduction

Coral reefs serve as a living environment for more than 30% of marine animals and plants [1, 2], while they are suffering from a sharp decline due to global warming, changes in the physicochemical environment of the ocean, and massive encroachment of the predatory crown-of-thorns starfish [3–7]. Several researchers even propose that features similar to those exhibited during the last mass extinction have emerged in scleractinian coral populations, including population shrinkage, transplanting of colonies to the aphotic zone, and zygote dormancy [8, 9]. Such a serious situation has brought about research focused on the growth, restoration, and ecological defense of reef-building corals.

However, in contrast to more common model organisms, there remains a lack of public omics data from reef-building corals, with no exception for the proteome bridging the physiological function and genome. Taking the UniProt database as an example, as of June 2022, it has collected no more than 50 reviewed proteins from *Acropora*, one of the most species-rich coral genera, while for another 2 dominant genera, *Montipora* and *Pocillopora*, the number even fails to reach 5. Various factors such as geographic location and technical limitations result in the “data gap” for coral research [10], and to make matters worse, COVID-19 has exacerbated the risks of sampling in the wild as well as performing “wet” experiments. Mass spectrometry technology having been applied in coral proteomics research [11–14] enables researchers to obtain high-throughput protein information from relatively small samples in standardized steps, yet according to traditional protocols, downstream analysis of protein structures and functions still requires cumbersome manual operations.

The booming artificial intelligence (AI) technology is expected to provide new solutions for the current predicament. The results of the biennial Critical Assessment of protein Structure Prediction (CASP) have revealed the substantial progress in protein structure prediction [15]. In CASP14 (2020), AlphaFold2 [16] achieving a record score of 92.4 was brought to the spotlight. As the structure modeling solution challenging x-ray crystallography and cryo-electron microscopy, AlphaFold2 can directly transform sequences to structures with high accuracy, which is particularly beneficial for studies on several nonmodel organisms represented by corals. These species may be important to our ecology and society, but existing experimental protocols for them are not as perfect as those for model organisms; moreover, people tend to focus on their potentially valuable components rather than a head-to-tail understanding of them. Assisted by AI modeling, scientists can rapidly acquire their structural proteomes [17] in the digital lab, then use computational biology methods to find key proteins and explore crucial physiological functions, so as to improve genome annotation and pave the way for applications, including breeding and protection.

Compared to other prediction algorithms such as RoseTTAFold [18], AlphaFold2 has obvious disadvantages in performance [19], as even protein domains can consume long computation times [20]. Fortunately, its open source has attracted joint efforts of developers to improve it. In 2021, Zhong and colleagues, from the Center for High Performance Computing (HPC) at Shanghai Jiao Tong University, released ParaFold [21], the specific AlphaFold version for their HPC clusters, making a successful attempt to accelerate AlphaFold2. In May 2022, ColabFold [22], which claimed to make protein folding accessible to all, was officially published. Replacing Jackhmmer with MMseqs2 [23] and utilizing an optimized model, ColabFold is able to run dozens of times faster than the original AlphaFold2, which means it greatly expands the throughput of protein structure modeling, thus breaking the last hurdle in effectively predicting the complete structural proteome.

Here we integrated mass spectrometry with the AI system to obtain digital structural proteomes of dominant reef-building corals. Deploying ColabFold in the Big Data Computing Center at Southeast University, we predicted structures of more than 8,000 three-way homologous proteins among *Acropora muricata, Montipora foliosa*, and *Pocillopora verrucosa* in approximately 4,060 GPU hours. The resulting dataset, named CP-8382, covers 83.6% of residues with a confident prediction and 25.9% with very high confidence. We also developed a search engine interface in the style of the AlphaFold Protein Structure Database [24] (AFDB for short) to open our data to the community (http://corals.bmeonline.cn/prot/).

## Materials and Methods

### Experimental model and subject details

The species including *A. muricata, M. foliosa*, and *P. verrucosa* in the study were collected from the Xisha Islands in the South China Sea (latitude 15°40′′–17°10′′ north, longitude 111°–113° east).

The coral samples were cultured in our laboratory coral tank with conditions conforming to their habitat environment. All the species were raised in a RedSea tank (redsea575; Red Sea Aquatics Ltd, Herzliya, Israel) at 26°C and 1.025 salinity (Red Sea Aquatics Ltd). The physical conditions of the coral culture system are as follows: 3 coral lamps (AI; Red Sea Aquatics Ltd), a protein skimmer (regal250s; Reef Octopus, Hong Kong), a water chiller (tk1000; TECO Ltd, Ravenna, Italy), 2 wave devices (VorTech MP40; EcoTech Marine Ltd, Bethlehem, United States of America), a calcium reactor (Calreact 200; Reef Octopus), and so on.

### Total protein extraction

Sample was ground individually in liquid nitrogen and lysed with PASP lysis buffer (100 mM NH_4_HCO_3_, 8 M urea, pH 8), followed by 5 minutes of ultrasonication on ice. The lysate was centrifuged at 12,000 × *g* for 15 minutes at 4°C and the supernatant was reduced with 10 mM DL-Dithiothreitol (DTT) for 1 hour at 56°C and subsequently alkylated with sufficient Iodoacetamide (IAM) for 1 hour at room temperature in the dark. Then samples were completely mixed with 4 times volume of precooled acetone by vortexing and incubated at −20°C for at least 2 hours. Samples were then centrifuged at 12,000 × *g* for 15 minutes at 4°C and the precipitation was collected. After washing with 1 ml cold acetone, the pellet was dissolved by dissolution buffer (8 M urea, 100 mM Triethylammonium bicarbonate (TEAB), pH 8.5).

### Protein quality test

Bovine serum albumin (BSA) standard protein solution was prepared according to the instructions of the Bradford protein quantitative kit, with gradient concentration ranging from 0 to 0.5 g/l. BSA standard protein solutions and sample solutions with different dilution multiples were added into the 96-well plate to fill the volume to 20 µl, respectively. Each gradient was repeated 3 times. In total, 180 μL G250 dye solution was quickly added to the plate and placed at room temperature for 5 minutes, and the absorbance at 595 nm was detected. The standard curve was drawn with the absorbance of standard protein solution, and the protein concentration of the sample was calculated. Then, 20 μg of the protein sample was loaded to 12% sodium dodecyl sulfate–polyacrylamide gel electrophoresis, wherein the concentrated gel was performed at 80 V for 20 minutes, and the separation gel was performed at 120 V for 90 minutes. The gel was stained by Coomassie brilliant blue R-250 and decolored until the bands were visualized clearly.

### Tandem Mass Tags (TMT) labeling of peptides

Each protein sample was taken and the volume was made up to 100 μL with DB dissolution buffer (8 M urea, 100 mM TEAB, pH 8.5). Trypsin and 100 mM TEAB buffer were added, and the sample was mixed and digested at 37°C for 4 hours. Then, trypsin and CaCl_2_ were added, and the sample was digested overnight. Formic acid was mixed with the digested sample, with an adjusted pH under 3, and centrifuged at 12,000 × *g* for 5 minutes at room temperature. The supernatant was slowly loaded to the C18 desalting column, washed with washing buffer (0.1% formic acid, 3% acetonitrile) 3 times, and then eluted by some elution buffer (0.1% formic acid, 70% acetonitrile). The eluents of each sample were collected and lyophilized. Then, 100 μL of 0.1 M TEAB buffer was added to reconstitute, and 41 μL of acetonitrile-dissolved TMT labeling reagent was added; the sample was mixed with shaking for 2 hours at room temperature. Then, the reaction was stopped by adding 8% ammonia. All labeling samples were mixed with equal volume, desalted, and lyophilized.

### Separation of fractions

Mobile phases A (2% acetonitrile, adjusted pH to 10.0 using ammonium hydroxide) and B (98% acetonitrile) were used to develop a gradient elution. The lyophilized powder was dissolved in solution A and centrifuged at 12,000 × *g* for 10 minutes at room temperature. The sample was fractionated using a C18 column (Waters, Milford, United States of America BEH C18, 4.6 × 250 mm, 5 μm) on a Rigol, Portland, United States of America L3000 HPLC system, and the column oven was set as 45°C. The detail of elution gradient is shown in Supplementary Table S1. The eluates were monitored at UV 214 nm, collected in a tube per minute, and combined into 10 fractions finally. All fractions were dried under vacuum and then reconstituted in 0.1% (v/v) formic acid in water.

### Liquid chromatography/tandem mass spectrometry analysis

For transition library construction, shotgun proteomics analyses were performed using an EASY-nLC 1200 UHPLC system (Thermo Fisher, Waltham, United States of America) coupled with a Q Exactive series mass spectrometer (Thermo Fisher) operating in the data-dependent acquisition mode. Then, a 1-μg sample was injected into a homemade C18 Nano-Trap column (4.5 cm × 75 μm, 3 μm). Peptides were separated in a homemade analytical column (15 cm × 150 μm, 1.9 μm), using a linear gradient elution as listed in Supplementary Table S2. The separated peptides were analyzed by a Q Exactive series mass spectrometer (Thermo Fisher), with the Nanospray Flex (ESI: Electron Spray Ionization; Nanospray Flex manufacturer: Thermo Fisher) as an ion source, a spray voltage of 2.3 kV, and an ion transport capillary temperature of 320°C. Full scan ranged from *m/z* 350 to 1,500 with a resolution of 60,000 (at *m/z* 200), an automatic gain control (AGC) target value was 3 × 10^6^, and a maximum ion injection time was 20 ms. The top 40 precursors of the highest abundance in the full scan were selected and fragmented by higher energy collisional dissociation and analyzed with tandem mass spectrometry, where resolution was 45,000 (at *m/z* 200) for 10 plex, the AGC target value was 5 × 10^4^, the maximum ion injection time was 86 ms, a normalized collision energy was set as 32%, an intensity threshold was 1.2 × 10^5^, and the dynamic exclusion parameter was 20 seconds.

### Protein identification and quantitation

The resulting spectra from each run were searched separately against protein-coding sequences from NCBI Bioproject PRJNA544778 by Proteome Discoverer 2.2 (PD 2.2; Thermo Fisher) [25]. The searched parameters were set as follows: mass tolerance for precursor ion was 10 ppm, and mass tolerance for product ion was 0.02 Da. Carbamidomethyl was specified as a fixed modification, oxidation of methionine (M) and TMT plex were specified as dynamic modifications, and acetylation and TMT plex were specified as N-terminal modifications in PD 2.2. A maximum of 2 miscleavage sites were allowed.

In order to improve the quality of analysis results, the software PD 2.2 further filtered the retrieval results: peptide spectrum matches (PSMs) with a credibility of more than 99% were identified PSMs. The identified protein contains at least 1 unique peptide. The identified PSMs and protein were retained and performed with a false discovery rate (FDR) of no more than 1.0%.

### Structure modeling

Three-way homologous proteins in *A. muricata, M. foliosa*, and *P. verrucosa* were selected for structure modeling. Representative sequences of them were sorted by length and numbered as CPXXXXXXXX according to order before being sent to the ColabFold (1.3.0) platform. A total of 1,053 proteins no longer than 200 Amino acid (aa) were calculated on the NVIDIA (Santa Clara, CA, USA) Tesla P100 while others were calculated on the NVIDIA Tesla V100 cluster at the Big Data Computing Center of Southeast University. The parameters of ColabFold were set to *–amber, –templates, –num-recycle 3, –use-gpu-relax*. For each protein, the structure with the highest predicted local distance difference test (pLDDT) scores (*_relaxed_rank_1_model_x.pdb) was preserved and labeled as CPXXXXXXXX.pdb.

Four hundred structures predicted in this work were selected and aligned to public AlphaFold structures of their similar proteins (BLAST E value <2.8e-309) by PyMOL (RRID:SCR_000305), and then root mean square deviations (RMSDs) were calculated.

### Protein annotation

Each coral protein was annotated by NR (diamond v2.0.14.152, *blastp –evalue 1e-5 -k 1*), UniProt (same tool and parameter as NR), and InterPro (interproscan-5.54–87.0). Pfam annotations for them were retrieved via eggNOG-mapper (RRID:SCR_021165) [26].

Template search results generated by ColabFold (*.template_domain_names.json) were compared to SCOP2 [27] and CATH (CATH: Protein Structure Classification, RRID:SCR_007583) [28].

### Search engine development

Elasticsearch 7.12.1 was employed as the key module, where NR, UniProt, and InterPro annotations were transformed into keywords in the index. Web interface was implemented using PHP 7.2, while Nginx worked as the web server.

Sequenceserver [29] 2.0.0 was deployed as the BLAST server, the source codes of which were modified to link each hit to its information page. Mol* [30] in the style of AFDB was imported as a structure viewer.

## Results and Discussion

### Protein identification and annotation

In total, 8,382 three-way homologous proteins in *A. muricata, M. foliosa*, and *P. verrucosa* were identified by Proteome Discoverer, and their representative sequences and expression profiles are shown in Supplementary Table S3. It is assumed that a considerable portion of proteomes is conserved among these dominant coral genera, for the total protein-coding gene number of 1 scleractinian coral is unlikely to exceed 25,000 according to previous reports [9–10, 31]. Over 97% of them are shorter than 2,600 aa, while the longest is up to 14,622 aa.

**Figure 1:**
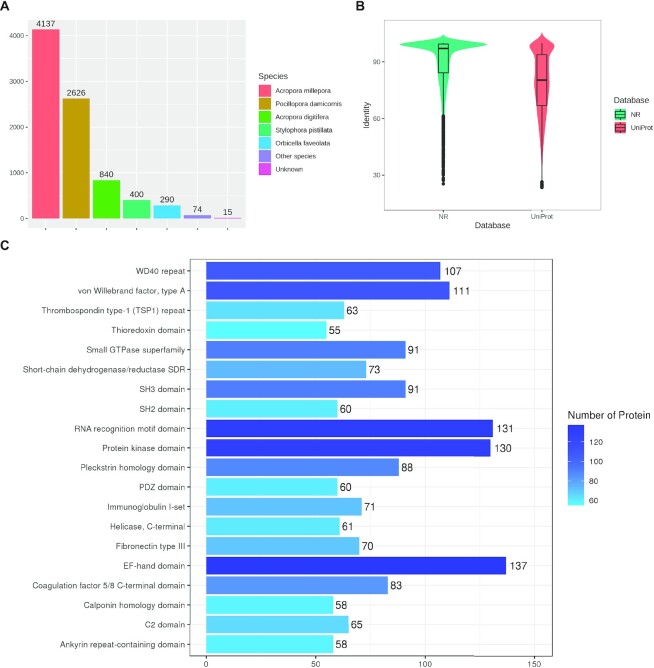
Statistics of coral protein annotations. (A) Homolog annotation with NR database. Horizontal axis is species and vertical axis is protein number. The top 5 species with the highest number of annotated proteins are *Acropora millepora, Pocillopora damicornis, Acropora digitifera, Stylophora pistillata*, and *Orbicella faveolata*. Only 15 proteins fail to get any NR annotations. (B) Violin plot of identity distribution. Horizontal axis is homolog database and vertical axis is identity. (C) Domain annotation with InterPro. Horizontal axis is protein number and vertical axis is domain name. Top 20 domains with largest counts are displayed.

Proteins were annotated with NR and UniProt for homologs and InterPro for domains. NR annotations map most proteins to scleractinians (Fig. 1A), rather than other cnidarians or marine organisms, with an average identity beyond 90% (Fig. 1B). It indicates the efforts into filling the “data gap” hindering coral research [10], as an increasing number of coral sequences have been added into public databases in recent years. Although according to Fig. 1B, coral sequences in UniProt may not be as abundant as NR, the 2 databases could complement each other to improve annotation. Fig. 1C demonstrates domains frequently found on coral proteins. EF-hand domain related to calcium signaling pathways [32] gets the top 1, consistent with these corals’ roles as reef builders, which deal with calcium ion every day for skeleton construction and homeostatic regulation. The RNA recognition motif domain and protein kinase domain are common protein domains in eukaryotes. von Willebrand factor (type A) may play an important role in coral immunity, as it has been reported to participate in allorejection responses [33]. WD40 repeats with a “doughnut hole” usually serve as protein interaction scaffolds in multiprotein complexes [34], which might also regulate innate immunity and stress response [35, 36].

Additionally, Pfam annotation of the coral proteome was made as a supplement to InterPro, and statistics of Pfam families are shown in Supplementary Fig. S1. All annotations are available in Supplementary Table S4.

### Protein structure prediction

**Figure 2:**
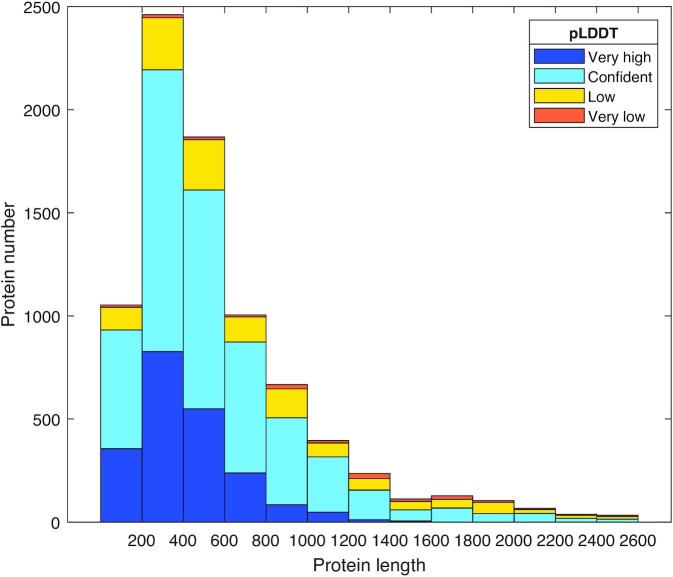
Distribution of model confidence against protein length. Horizontal axis is protein length and vertical axis is protein number. Model confidence calculated by pLDDT is color-coded. Very high: pLDDT > 90; confident: 90 > pLDDT > 70; low: 70 > pLDDT > 50; very low: pLDDT < 50.

After about 4,060 GPU hours of computation, ColabFold succeeded to generate 8,166 structures, covering 97.4% of our coral protein dataset and touching the maximum length that an NVIDIA Tesla V100 (32 GB) can process. The model confidence distribution is presented in Fig. 2. In the resulting dataset, 83.6% of residues had a pLDDT larger than 70, which are considered confident predictions [15], and 25.9% had a very high pLDDT over 90. No more than 2% of predicted structures had an average pLDDT below 50. The predictions from ColabFold can be recognized as credible overall.

An attempt was made to compare the predicted structure models to a public protein structural classification database. ColabFold found 27,887 template domains, among which nearly 70% have been registered in SCOP or CATH (Supplementary Fig. S2). Nevertheless, current template search results have turned out to be sequence based, not final structure based; thus, using them to represent fold distributions might fail to avoid false positives. It would be better to apply high-throughput structure alignment methods into the classification of remaining template domains as well as annotation of various novel folds, yet existing methods are not efficient enough to handle the increasing scale of data pushed from AI systems.

Differences between AlphaFold and ColabFold predictions were also observed. Four hundred structures in the resulting dataset were aligned to public AlphaFold structures of their similar proteins, and then RMSDs were calculated. As illustrated in Supplementary Fig. S3, most structure pairs seem to have little difference, but some do differ significantly. Considering the lack of exactly the same coral proteins in AFDB, it might be difficult to detect whether the sequential feature or the AI system itself is responsible for those differences. Hence, until more AlphaFold-registered or experimentally verified structures are available, pLDDT will remain the main technical control indicator for coral protein structure prediction; meanwhile, the resulting dataset of this work may be able to temporarily serve as a coral-specific extension of AFDB for RMSD analysis.

### Highlighted predictions

**Figure 3:**
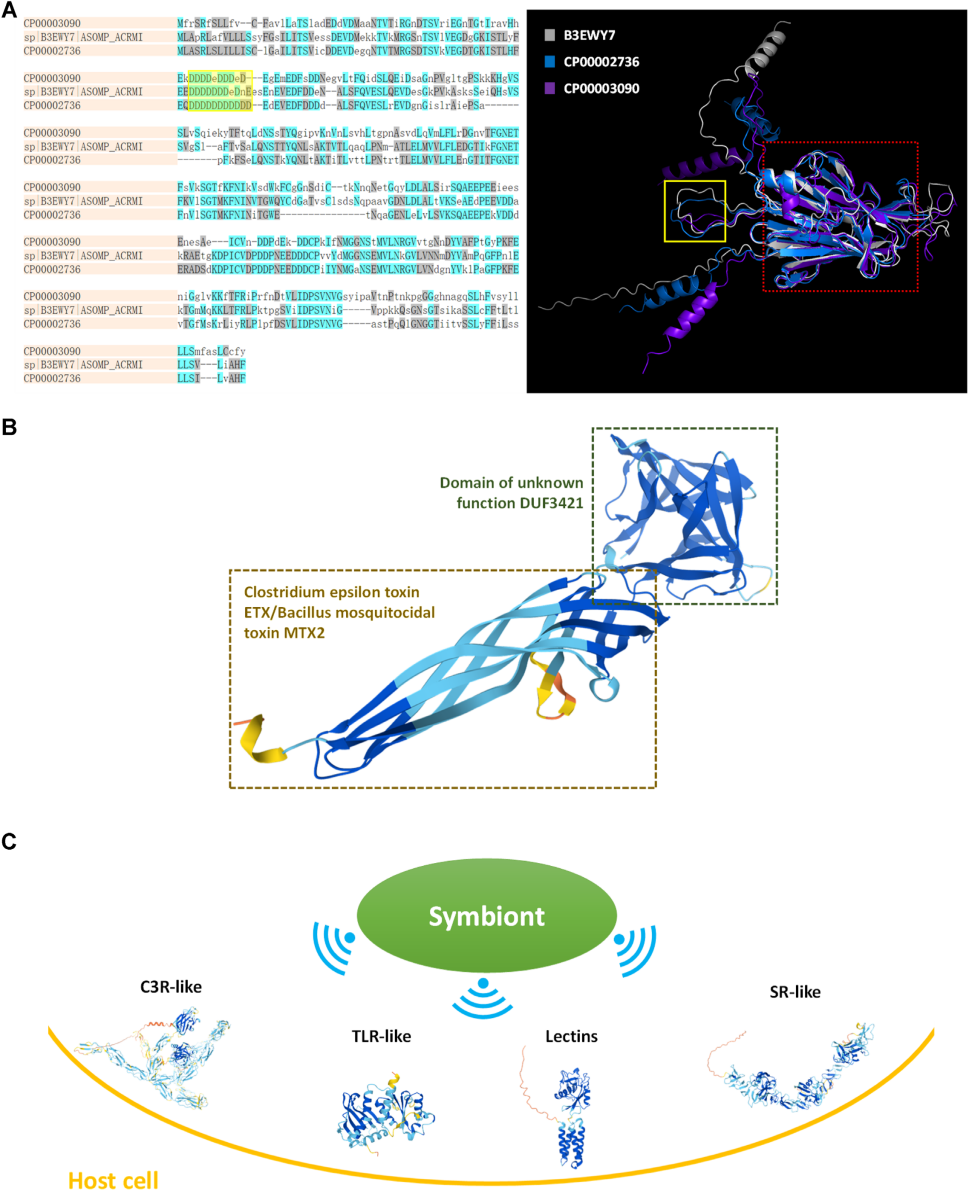
Highlighted structure predictions. (A) Sequence and structure alignment among CP00002736, CP00003090, and B3EWY7. Asp-rich regions are framed in yellow and β-sheet-formed regions are framed in red. (B) Structure of CP00002607, similar to natterin 4. (C) Structures of several pattern recognition receptors involved in host–symbiont signaling, including C3R (complement 3 receptor), TLR (Toll-like receptor), lectin, and SR (scavenger receptor).

Fig. 3 demonstrates several highlighted structure predictions from the resulting dataset. There are few doubts that biomineralization is the most significant function of reef-building corals [37, 38]; thus, their skeletal proteome regulating the mineral deposition is always of interest to scientists. Our results cover most of recognized coral mineralization-related proteins, including skeletal organic matrix protein (SOMP), skeletal aspartic acid–rich protein, collagen, carbonic anhydrase, and so on [11, 12]. Fig. 3A shows the sequence and structure alignment among 3 acidic SOMPs, 2 identified in this work and 1 from UniProt (B3EWY7). Although the sequence identity is only 40% to 60%, it appears that their structures are broadly similar, consisting of an Asp-rich tongue-like region and a β-sheet-formed region. According to previous studies, Asp-rich proteins are supposed to interact directly with calcium carbonate crystals promoting crystal nucleation, determining the growth axes and inhibiting the crystal growth [39, 40], and they usually have high-capacity yet low-affinity calcium-binding properties [41]. Our structure predictions may provide an explanation that for these proteins, Asps are concentrated in an open tongue-like region, reducing the steric hindrance while weakening the binding strength with calcium ions. Besides, the β-sheet-rich region at the base of the “Asp tongue” forms a barrel-like local structure, which is also found in many other uncharacterized skeletal organic matrix proteins (USOMPs) (Supplementary Fig. S4). These barrel-like regions probably have transmembrane functions [42], but existing methods fail to annotate them with any known domains, making these SOMPs uncharacterized. This phenomenon might not only urge biologists to gain deeper insights into protein domains but also enlighten bioinformaticians to design novel annotation algorithms for 3-dimensional structure data.

Toxins are widely employed by marine organisms for prey capture and defense of the territory [43]. Natterin proteins discovered in the venom of the medically significant Brazilian toadfish *Thalassophryne nattereri* [44] are representative perforin-like toxins that can insert into the lipid bilayer to trigger the disruption of membrane function [45]. We identify 1 natterin 4–like protein, CP00002607, as illustrated in Fig. 3B. The drill-like toxic domain is supposed to “bore holes” on membranes, resulting in electrolyte leakage and inflammation [46]. The function of DUF3421 has not been commonly recognized [47], although several researchers propose that such DM9-containing proteins are new members of pattern recognition receptors (PRRs) [48–50]. From a structural point of view, this DM9-containing domain is at the end of a “toxic drill” with a funnel-like cavity. It indeed has potential to bind signaling molecules, playing roles in the antagonism between toxin and the immune system; nevertheless, the possibility that it just exacerbates membrane damage or attaches other toxic chemicals cannot be ruled out. This structure prediction might help to deepen our understanding of marine biotoxins and provide inspiration for drug development [51], not to mention that it can serve as raw input for molecular docking itself.

Symbiosis is another important topic for coral biologists and ecologists, as reef-building corals obtain the majority of their energy and nutrients from their algal symbionts (mainly Symbiodiniaceae [52]), and loss of symbionts is causing coral bleaching, threatening marine ecology. Improved knowledge of interpartner signaling in coral holobionts could be applied to solutions against the coral reef crisis [53]. Previous studies reveal that the initiation of coral symbiosis depends on the interaction between PRRs on host gastrodermal nutritive phagocytes and microbe-associated molecular patterns (MAMPs) of symbiont [54, 55], as shown in Fig. 3C. Complete structures of several representative PRRs, such as complement 3 receptor, Toll-like receptor, lectin, and scavenger receptor, get confident prediction in our work, with a pLDDT ranging from 75 to 88. Unfortunately, our predictions fail to cover PRRs too large for our GPU devices to process, and symbiont proteins including MAMPs are beyond the scope of our experiment design. It will be an excellent work to acquire Symbiodiniaceae structural proteomes and integrate them with corals, which may bring about novel insights into coral symbiosis as well as interpartner signaling in cnidarians. In fact, the “data gap” for symbionts in coral holobionts turns out to be even larger than that for corals themselves [10], presenting both challenges and opportunities.

### Web interface of CP-8382

**Figure 4:**
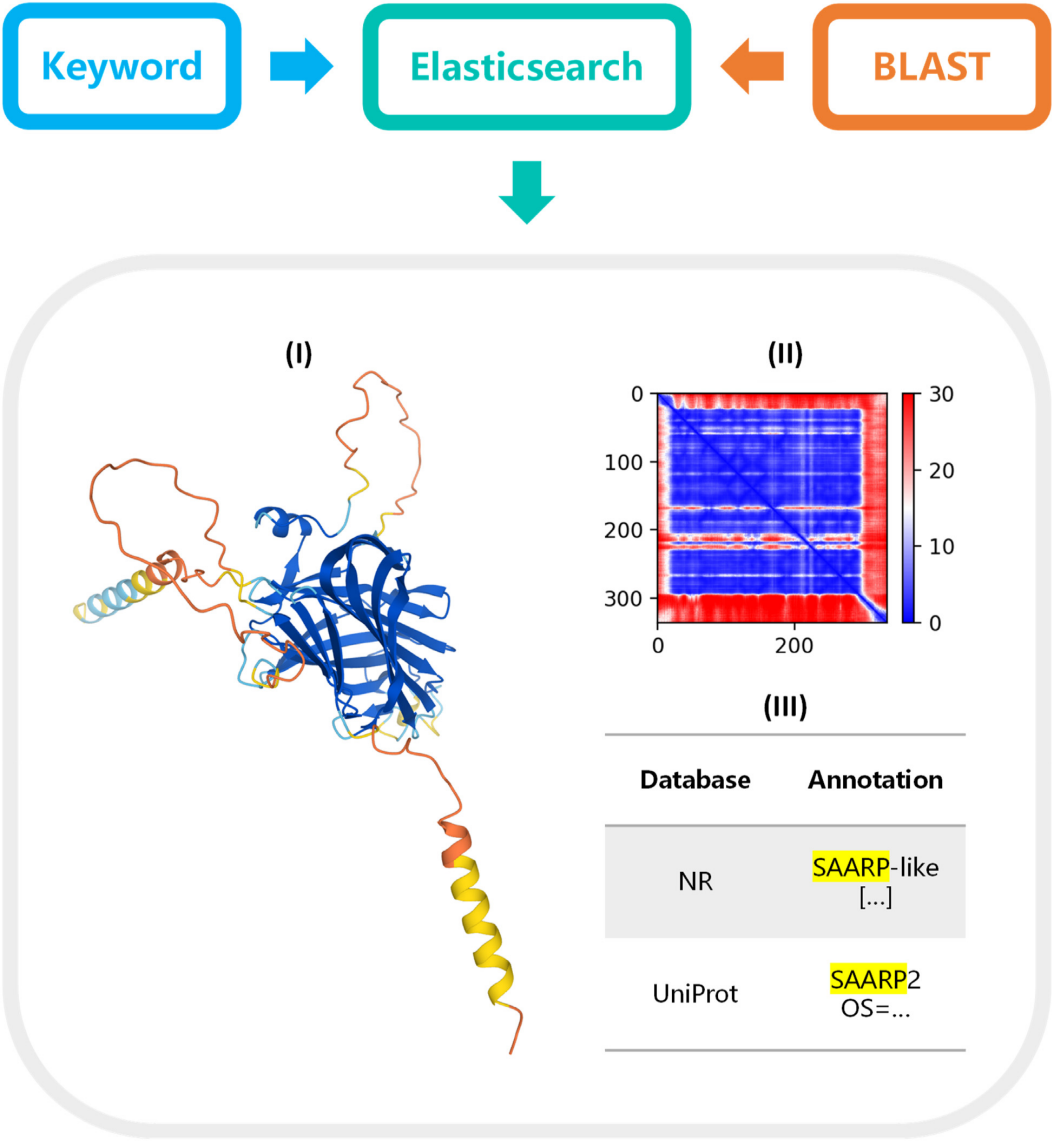
Content of CP-8382 dataset and workflow of its web interface. Users can utilize search engine or BLAST server to acquire their interested coral proteins on the website, where the elasticsearch module will process inputs and return the following information: (I) predicted structure displayed in the style of AFDB; (II) PAE graph, where the color at position (x, y) indicates AlphaFold's expected position error at residue x when the predicted and true structures are aligned on residue y; and (III) annotations with keywords highlighted.

Combining annotations and predicted structures, the resulting dataset was named CP-8382 and curated into an online database [57]. Fig. 4 shows its data content as well as the workflow of the corresponding web interface. Predicted structures are displayed in the style of AFDB, where the pLDDT of each residue is marked by color, and Mol* app enables users to zoom, rotate, or take a screenshot. They can be downloaded in the format of PDB or CIF. Predicted aligned error (PAE) graphs are given for assessing confidence in global features [15], raw data of which are available in the JSON format. Annotations with keywords highlighted are also presented. All the above information is accessible via search engine or BLAST server at the web interface. Supplementary Video S1 provides a demo of searching skeletal aspartic acid–rich protein in CP-8382.

## Conclusion

By the aid of ColabFold, we succeeded to generate a content-rich structure dataset for reef-building corals within an acceptable period. Its claim to make protein folding accessible to all is seemingly not an exaggeration; furthermore, the relatively high confidence of our results might prove that ColabFold does not sacrifice too much accuracy for speed [22].

Our work is expected to be not only a contribution to coral research but also an early case of digital structural proteome building. Computational biologists preferring similar approaches may optimize their resource allocation by referring to our experience. Moreover, moving most preliminary work to a “dry lab” will both accelerate research progress via HPC technology and boost peer communication through the Internet, so as to facilitate problem solving in a more efficient and economical way. In view that the open source of RoseTTAFold and AlphaFold2 has brought the dawn of high-throughput structural proteomics, we recommend researchers, especially those interested in not famous yet potentially important organisms, to build and publish digital structural proteomes, which is conducive to new solutions from a structural perspective, people's deeper understanding of biodiversity, and attraction of joint efforts.

## Additional Files

**Supplementary Figure S1**. Statistics of Pfam annotation. Horizontal axis is protein number and vertical axis is Pfam family name. Top 50 Pfam families with largest counts are displayed.

**Supplementary Figure S2**. Venn plot of template domain distribution among ColabFold search results, SCOP and CATH.

**Supplementary Figure S3**. Statistics of RMSDs between ColabFold and AlphaFold predictions.

**Supplementary Figure S4**. Structures of USOMP-like proteins. Regions shaped like β-barrels are marked with red boxes.

**Supplementary Table S1**. Peptide fraction separation with a liquid chromatography elution gradient.

**Supplementary Table S2**. Liquid chromatography elution gradient.

**Supplementary Table S3**. Sequences and expression profiles of identified proteins.

**Supplementary Table S4**. Model scores, annotations, and template domains of identified proteins.

**Supplementary Table S5**. RMSDs between ColabFold and AlphaFold predictions.

**Supplementary Video S1**. Demo of searching skeletal aspartic acid–rich protein via web interface of CP-8382.

giac117_GIGA-D-22-00178_Original_Submission

giac117_GIGA-D-22-00178_Revision_1

giac117_GIGA-D-22-00178_Revision_2

giac117_GIGA-D-22-00178_Revision_3

giac117_Response_to_Reviewer_Comments_Original_Submission

giac117_Response_to_Reviewer_Comments_Revision_1

giac117_Response_to_Reviewer_Comments_Revision_2

giac117_Reviewer_1_Report_Original_Submission Jianyi Yang -- 8/31/2022 Reviewed

giac117_Reviewer_1_Report_Revision_1 Jianyi Yang -- 10/4/2022 Reviewed

giac117_Reviewer_2_Report_Original_Submission Brendan Robert E. Ansell, PhD -- 9/26/2022 Reviewed

giac117_Reviewer_2_Report_Revision_1 Brendan Robert E. Ansell, PhD -- 10/5/2022 Reviewed

giac117_Reviewer_2_Report_Revision_2 Brendan Robert E. Ansell, PhD -- 10/6/2022 Reviewed

giac117_Supplemental_Files

## Data Availability

The mass spectrometry proteomics data have been deposited to the ProteomeXchange Consortium via the iProX partner repository [56] with the dataset identifier PXD034973.

All supporting data and materials are available in the *GigaScience* GigaDB database [57].

## Abbreviations

AFDB: AlphaFold Protein Structure Database; AGC: automatic gain control; AI: artificial intelligence; BLAST: Basic Local Alignment Search Tool; BSA: bovine serum albumin; CASP: Critical Assessment of protein Structure Prediction; HPC: high-performance computing; MAMP: microbe-associated molecular pattern; NCBI: The National Center for Biotechnology Information; PAE: predicted aligned error; pLDDT: predicted local distance difference test; PRR: pattern recognition receptor; PSM: peptide spectrum match; RMSD: root mean square deviation; SOMP: skeletal organic matrix protein; USOMP: uncharacterized skeletal organic matrix protein.

## Competing Interests

The authors declare that they have no competing interests.

## Ethics Approval and Consent to Participate

All coral samples were collected and processed in accordance with local laws for invertebrate protection and approved by the Ethics Committee of Institutional Animal Care and Use Committee of Nanjing Medical University (protocol code IACUC-1910003, and date of approval is 10 October 2019).

## Authors' Contributions

Y.Z.: experiment, database, writing, and editing. T.H.: data uploading. Z.L. and J.C.: reviewing. C.H.: supervision. X.L.: project approval. All authors contributed to the article and approved the submitted version.

## Funding

This work was supported by the open research fund of State Key Laboratory of Bioelectronics, Southeast University (Sklb2021-k02) and the open research fund program of Guangxi Key Lab of Mangrove Conservation and Utilization (GKLMC-202002).

## References

[1] Odum HT, Odum EP. Trophic structure and productivity of a windward coral reef community on Eniwetok Atoll. Ecological Monographs. 1955;25(3):291–320.

[2] Yu KF . Coral reefs in the South China Sea: their response to and records on past environmental changes. Sci China Earth Sci. 2012;55(8):1217–29.

[3] Moberg F, Folke C. Ecological goods and services of coral reef ecosystems. Ecol Econ. 1999;29(2):215–33.

[4] Wilson SK, Graham NA, Pratchett MS, et al. Multiple disturbances and the global degradation of coral reefs: are reef fishes at risk or resilient?. Global Change Biol. 2006;12(11):2220–34.

[5] Nakamura M, Okaji K, Higa Y, et al. Spatial and temporal population dynamics of the crown-of-thorns starfish, Acanthaster planci, over a 24-year period along the central west coast of Okinawa Island. Jpn Marine Biol. 2014;161(11):2521–30.

[6] Reimer JD, Kise H, Wee HB, et al. Crown-of-thorns starfish outbreak at oceanic Dongsha Atoll in the northern South China Sea. Mar Biodivers. 2019;49(6):2495–7.

[7] Magel JMT, Dimoff SA. Direct and indirect effects of cimate change-amplified pulse heat stress events on coral reef fish communities. Bull Ecol Soc Am. 2020;101(3):1–6. 10.1002/eap.2124 32167633

[8] Dishon G, Grossowicz M, Krom M, et al. Evolutionary traits that enable scleractinian corals to survive mass extinction events. Sci Rep. 2020;10(1):3903. 32127555 10.1038/s41598-020-60605-2 PMC7054358

[9] Guo Z, Liao X, Chen JY, et al. Binding pattern reconstructions of FGF-FGFR budding-inducing signaling in reef-building corals. Front Physiol. 2022;12:759370. 35058792 10.3389/fphys.2021.759370 PMC8764167

[10] Zhu Y, Liao X, Han T, et al. Symbiodiniaceae microRNAs and their targeting sites in coral holobionts: a transcriptomics-based exploration. Genomics. 2022;114(4):110404. 35714829 10.1016/j.ygeno.2022.110404

[11] Ramos-Silva P, Kaandorp J, Huisman L, et al. The skeletal proteome of the coral Acropora millepora: the evolution of calcification by co-option and domain shuffling. Mol Biol Evol. 2013;30(9):2099–112. 23765379 10.1093/molbev/mst109 PMC3748352

[12] Drake JL, Mass T, Haramaty L, et al. Proteomic analysis of skeletal organic matrix from the stony coral Stylophora pistillata. Proc Natl Acad Sci U S A. 2013;110(10):3788–93. 23431140 10.1073/pnas.1301419110 PMC3593878

[13] Conci N, Lehmann M, Vargas S, et al. Comparative proteomics of octocoral and scleractinian skeletomes and the evolution of coral calcification. Genome Biol Evol. 2020;12(9):1623–35. 32761183 10.1093/gbe/evaa162 PMC7533068

[14] Peled Y, Drake JL, Malik A, et al. Optimization of skeletal protein preparation for LC-MS/MS sequencing yields additional coral skeletal proteins in Stylophora pistillata. BMC Mater. 2020;2:8. 32724895 10.1186/s42833-020-00014-x PMC7115838

[15] Tunyasuvunakool K, Adler J, Wu Z, et al. Highly accurate protein structure prediction for the human proteome. Nature. 2021;596(7873):590–6. 34293799 10.1038/s41586-021-03828-1 PMC8387240

[16] Jumper J, Evans R, Pritzel A, et al. Highly accurate protein structure prediction with AlphaFold. Nature. 2021;596(7873):583–9. 34265844 10.1038/s41586-021-03819-2 PMC8371605

[17] Yee A, Pardee K, Christendat D, et al. Structural proteomics: toward high-throughput structural biology as a tool in functional genomics. Acc Chem Res. 2003;36(3):183–9. 12641475 10.1021/ar010126g

[18] Baek M, DiMaio F, Anishchenko I, et al. Accurate prediction of protein structures and interactions using a three-track neural network. Science. 2021;373(6557):871–6. 34282049 10.1126/science.abj8754 PMC7612213

[19] Cheng S, Wu R, Yu Z et al. FastFold: reducing AlphaFold training time from 11 days to 67 hours. 2022.abs/2203.00854. doi:10.48550/arXiv.2203.00854.

[20] Zhu Y, Lu N, Chen JY, et al. Deep whole-genome resequencing sheds light on the distribution and effect of amphioxus SNPs. BMC Genom Data. 2022;23(1):26. 35395709 10.1186/s12863-022-01038-w PMC8994340

[21] Zhong B, Su X, Wen M, et al. ParaFold: paralleling AlphaFold for large-scale predictions. Presented at: International Conference on High Performance Computing in Asia-Pacific Region Workshops; January 2022:1–9. doi:10.1145/3503470.3503471.

[22] Mirdita M, Schütze K, Moriwaki Y, et al. ColabFold: making protein folding accessible to all. Nat Methods. 2022;19(6):679–82. 35637307 10.1038/s41592-022-01488-1 PMC9184281

[23] Mirdita M, Steinegger M, Söding J. MMseqs2 desktop and local web server app for fast, interactive sequence searches. Bioinformatics. 2019;35(16):2856–8. 30615063 10.1093/bioinformatics/bty1057 PMC6691333

[24] David A, Islam S, Tankhilevich E, et al. The AlphaFold database of protein structures: a biologist's guide. J Mol Biol. 2022;434(2):167336. 34757056 10.1016/j.jmb.2021.167336 PMC8783046

[25] Orsburn BC . Proteome Discoverer—a community enhanced data processing suite for protein informatics. Proteomes. 2021;9(1):15. 33806881 10.3390/proteomes9010015 PMC8006021

[26] Cantalapiedra CP, Hernández-Plaza A, Letunic I, et al. eggNOG-mapper v2: functional annotation, orthology assignments, and domain prediction at the metagenomic scale. Mol Biol Evol. 2021;38(12):5825–9. 34597405 10.1093/molbev/msab293 PMC8662613

[27] Andreeva A, Kulesha E, Gough J, et al. The SCOP database in 2020: expanded classification of representative family and superfamily domains of known protein structures. Nucleic Acids Res. 2020;48(D1):D376–82. 31724711 10.1093/nar/gkz1064 PMC7139981

[28] Sillitoe I, Bordin N, Dawson N, et al. CATH: increased structural coverage of functional space. Nucleic Acids Res. 2021;49(D1):D266–73. 33237325 10.1093/nar/gkaa1079 PMC7778904

[29] Priyam A, Woodcroft BJ, Rai V, et al. Sequenceserver: a modern graphical user interface for custom BLAST databases. Mol Biol Evol. 2019;36(12):2922–4. 31411700 10.1093/molbev/msz185 PMC6878946

[30] Sehnal D, Bittrich S, Deshpande M, et al. Mol* Viewer: modern web app for 3D visualization and analysis of large biomolecular structures. Nucleic Acids Res. 2021;49(W1):W431–7. 33956157 10.1093/nar/gkab314 PMC8262734

[31] Shinzato C, Khalturin K, Inoue J, et al. Eighteen coral genomes reveal the evolutionary origin of Acropora strategies to accommodate environmental changes. Mol Biol Evol. 2021;38(1):16–30. 32877528 10.1093/molbev/msaa216 PMC7783167

[32] Nelson MR, Thulin E, Fagan PA, et al. The EF-hand domain: a globally cooperative structural unit. Protein Sci. 2002;11(2):198–205. 11790829 10.1110/ps.33302 PMC2373453

[33] Oren M, Amar KO, Douek J, et al. Assembled catalog of immune-related genes from allogeneic challenged corals that unveils the participation of vWF-like transcript. Dev Comp Immunol. 2010;34(6):630–7. 20080125 10.1016/j.dci.2010.01.007

[34] Schapira M, Tyers M, Torrent M, et al. WD40 repeat domain proteins: a novel target class? Nat Rev Drug Discov. 2017;16(11):773–86. 29026209 10.1038/nrd.2017.179 PMC5975957

[35] Kong D, Li M, Dong Z, et al. Identification of TaWD40D, a wheat WD40 repeat-containing protein that is associated with plant tolerance to abiotic stresses. Plant Cell Rep. 2015;34(3):395–410. 25447637 10.1007/s00299-014-1717-1

[36] Liu WC, Li YH, Yuan HM, et al. WD40-REPEAT 5a functions in drought stress tolerance by regulating nitric oxide accumulation in Arabidopsis. Plant Cell Environ. 2017;40(4):543–52. 26825291 10.1111/pce.12723

[37] Wang X, Zoccola D, Liew YJ, et al. The evolution of calcification in reef-building corals. Mol Biol Evol. 2021;38(9):3543–55. 33871620 10.1093/molbev/msab103 PMC8382919

[38] Von Euw S, Zhang Q, Manichev V, et al. Biological control of aragonite formation in stony corals. Science. 2017;356(6341):933–8. 28572387 10.1126/science.aam6371

[39] Wheeler AP, George JW, Evans CA. Control of calcium carbonate nucleation and crystal growth by soluble matrx of oyster shell. Science. 1981;212(4501):1397–8. 17746262 10.1126/science.212.4501.1397

[40] Addadi L, Moradian J, Shay E, et al. A chemical model for the cooperation of sulfates and carboxylates in calcite crystal nucleation: Relevance to biomineralization. Proc Natl Acad Sci U S A. 1987;84(9):2732–6. 16593827 10.1073/pnas.84.9.2732 PMC304732

[41] Maurer P, Hohenester E, Engel J. Extracellular calcium-binding proteins. Curr Opin Cell Biol. 1996;8(5):609–17. 8939653 10.1016/s0955-0674(96)80101-3

[42] Fairman JW, Noinaj N, Buchanan SK. The structural biology of β-barrel membrane proteins: a summary of recent reports. Curr Opin Struct Biol. 2011;21(4):523–31. 21719274 10.1016/j.sbi.2011.05.005 PMC3164749

[43] Lima C, Disner GR, Falcão MAP, et al. The natterin proteins diversity: a review on phylogeny, structure, and immune function. Toxins (Basel). 2021;13(8):538. 34437409 10.3390/toxins13080538 PMC8402412

[44] Magalhães GS, Junqueira-de-Azevedo IL, Lopes-Ferreira M, et al. Transcriptome analysis of expressed sequence tags from the venom glands of the fish Thalassophryne nattereri. Biochimie. 2006;88(6):693–9. 16488069 10.1016/j.biochi.2005.12.008

[45] Dal Peraro M, van der Goot FG. Pore-forming toxins: ancient, but never really out of fashion. Nat Rev Microbiol. 2016;14(2):77–92. 26639780 10.1038/nrmicro.2015.3

[46] Greaney AJ, Leppla SH, Moayeri M. Bacterial exotoxins and the inflammasome. Front Immunol. 2015;6:570. 26617605 10.3389/fimmu.2015.00570 PMC4639612

[47] Ponting CP, Mott R, Bork P, et al. Novel protein domains and repeats in Drosophila melanogaster: insights into structure, function, and evolution. Genome Res. 2001;11(12):1996–2008. 11731489 10.1101/gr.198701

[48] Unno H, Matsuyama K, Tsuji Y, et al. Identification, characterization, and x-ray crystallographic analysis of a novel type of mannose-specific lectin CGL1 from the Pacific oyster Crassostrea gigas. Sci Rep. 2016;6:29135. 27377186 10.1038/srep29135 PMC4932603

[49] Jiang S, Wang L, Huang M, et al. DM9 domain containing protein functions as a pattern recognition receptor with broad microbial recognition spectrum. Front Immunol. 2017;8:1607. 29238341 10.3389/fimmu.2017.01607 PMC5712788

[50] Wang W, Song X, Wang L, et al. Pathogen-derived carbohydrate recognition in molluscs immune defense. Int J Mol Sci. 2018;19(3):721. 29510476 10.3390/ijms19030721 PMC5877582

[51] Li Y, Orange JS. Degranulation enhances presynaptic membrane packing, which protects NK cells from perforin-mediated autolysis. PLoS Biol. 2021;19(8):e3001328. 34343168 10.1371/journal.pbio.3001328 PMC8330931

[52] LaJeunesse TC, Parkinson JE, Gabrielson PW, et al. Systematic revision of Symbiodiniaceae highlights the antiquity and diversity of coral endosymbionts. Curr Biol. 2018;28(16):2570–80.e6. 30100341 10.1016/j.cub.2018.07.008

[53] Rosset SL, Oakley CA, Ferrier-Pagès C, et al. The molecular language of the cnidarian-dinoflagellate symbiosis. Trends Microbiol. 2021;29(4):320–33. 33041180 10.1016/j.tim.2020.08.005

[54] Davy SK, Allemand D, Weis VM. Cell biology of cnidarian-dinoflagellate symbiosis. Microbiol Mol Biol Rev. 2012;76(2):229–61. 22688813 10.1128/MMBR.05014-11 PMC3372257

[55] Weis VM . Cell biology of coral symbiosis: foundational study can inform solutions to the coral reef crisis. Integr Comp Biol. 2019;59(4):845–55. 31150064 10.1093/icb/icz067

[56] Ma J, Chen T, Wu S, et al. iProX: an integrated proteome resource. Nucleic Acids Res. 2019;47(D1):D1211–7. 30252093 10.1093/nar/gky869 PMC6323926

[57] Zhu Y, Liao X, Han T et al. Supporting data for “Utilizing artificial intelligence system to build the digital structural proteome of reef-building corals.”. GigaScience Database. 2022. 10.5524/102332 PMC9673494 36399057

