# Supplementary material for: Utilizing an artificial intelligence system to build the digital structural proteome of reef-building corals
Source: Gigascience. 2022 Nov 18;11:giac117. doi: 10.1093/gigascience/giac117 (PMC9673494; doi:10.1093/gigascience/giac117)
Supplement: giac117_GIGA-D-22-00178_Revision_1 [file giac117_giga-d-22-00178_revision_1.pdf]

GigaScience

Utilizing artificial intelligence system to build the digital structural proteome of reef-building corals

--Manuscript Draft--

**Manuscript Number:**	GIGA-D-22-00178R1
**Full Title:**	Utilizing artificial intelligence system to build the digital structural proteome of reef-building corals
**Article Type:**	Data Note
**Funding Information:**	open research fund of State Key Laboratory of Bioelectronics (Sklb2021-k02) — Prof. Chunpeng He<br>open research fund program of Guangxi Key Lab of Mangrove Conservation and Utilization (GKLMC-202002) — Prof. Xin Liao
**Abstract:**	Background Reef-building corals play an important role in the marine ecosystem, and analyzing their proteomes from a structural perspective will exert positive effects on exploring their biology. Here we integrated mass spectrometry with newly published ColabFold to obtain digital structural proteome s of dominant reef-building corals. Results 8,382 proteins co-expressed in A. muricata , M. foliosa and P. verrucosa were identified, then 8,166 of them got predicted structures after around 4,060 GPU hours of computation. The resulting dataset covers 83.6% of residues with a confident prediction, while 25.9% have very high confidence. Conclusions Our work provides insight-worthy predictions for coral research, confirms the reliability of ColabFold in practice, and is expected to be a reference case in the impending high-throughput era of structural proteomics.
**Corresponding Author:**	Yunchi Zhu<br>Southeast University<br>Nanjing, Jiangsu CHINA
**Corresponding Author Secondary Information:**	
**Corresponding Author's Institution:**	Southeast University
**Corresponding Author's Secondary Institution:**	
**First Author:**	Yunchi Zhu
**First Author Secondary Information:**	
**Order of Authors:**	Yunchi Zhu<br>Xin Liao<br>Tingyu Han<br>J.-Y. Chen<br>Chunpeng He<br>Zuhong Lu
**Order of Authors Secondary Information:**	
**Response to Reviewers:**	Dear Professor Zhou,<br><br>Thank you very much for giving us an opportunity to revise our manuscript Utilizing artificial intelligence system to build the digital structural proteome of reef-building corals (GIGA-D-22-00178). We feel obliged to express our great appreciation to all the editors and reviewers for the valuable comments and suggestions. We have carefully revised and supplemented our manuscript according to each comment, and all corrections are marked with different colours.

Powered by Editorial Manager® and ProduXion Manager® from Aries Systems Corporation

The main corrections in the paper and the responses to the comments are as following:

Reviewer #1:
1. Response to comment: I would encourage the authors to spend more effort in analyzing the sequences.
Thank you for this important reminder. Although it might not be required for the data note category, bioinformatics analysis will undoubtedly enhance the value of the publication. We have supplemented Pfam annotations (new Fig S1) as well as more sequence analyses (new Fig 1).

2. Response to comment: It may be meaningful to compare the predicted structure models to the SCOP or CATH database to see the fold distribution and if there is any new fold.
We have made an attempt to compare the predicted structure models to SCOP and CATH. ColabFold totally found 27,887 template domains, among which nearly 70% have been registered in SCOP or CATH (new Fig S2). Nevertheless, current template search results generated by ColabFold or AlphaFold turn out to be sequence-based, not final-structure-based, thus using them to represent fold distributions might fail to avoid false positives. From our perspective, it would be better to apply high-throughput structure alignment methods into classification of remaining template domains as well as annotation of various novel folds, yet existing methods seem to be not efficient enough to handle the increasing scale of data pushed from AI systems such as ColabFold.

3. Response to comment: What happened to the ~200 proteins that ColabFold failed to work?
ColabFold on Nvidia Tesla V100 (32G GPU memory) cannot process proteins larger than ~2550 AA. The maximum size of structure predictions is determined by GPU memory currently, even AlphaFold Protein Structure Database can only split large proteins (longer than 2700AA) into 1400AA fragments for structure prediction (https://www.alphafold.ebi.ac.uk/download). Since predicting structures of long fragments of the remaining 216 proteins (933,369 residues, average length 4,321 AA) may be time-consuming while one of our authors need the publication of this paper for his PhD, we fail to add this work to our revised manuscript, but we will gradually add these partial structure predictions to our web server. Besides, we are negotiating with colleagues to borrow a Nvidia Tesla A100 (80G GPU memory) for directly structure prediction of some long proteins.

4. Response to comment: I suggest adding a browse function to the server for browsing the data.
Browse function has been added at http://corals.bmeonline.cn/prot/browse.php.

Reviewer #2:
1. Response to comment: Please ensure that the entire structure repository is available for unrestricted download as per http://corals.bmeonline.cn/prot/release.php; The link from 'CoralBioinfo' gives a 404 error: http://corals.bmeonline.cn/index.php; In http://corals.bmeonline.cn/blast/, please include a link back to http://corals.bmeonline.cn/prot/.
Thank you for the suggestions above. All requirements have been completed.

2. Response to comment: Please replace 'co-expression' with 'homologous proteins'. Use full genus names on first use. Proofreading of grammar required throughout, and elimination of non-scientific phrasing.
Thank you for this important reminder. All incorrect terms have been corrected.

3. Response to comment: Although the manuscript lacks bioinformatic analysis of the structural proteome, this is not required for the data note category but would enhance the value of the publication if provided.
We have supplemented more sequence analyses (new Fig 1, new Fig S1) and made an attempt to compare the predicted structure models to SCOP and CATH (new Fig S2).

4. Response to comment: Please report the RMSD between structures predicted in this

work with the published alphafold structures for the same proteins.
From our experience, RMSD is usually calculated between different conformations of the same protein, while it is hard to find the same proteins (exactly same elements and same order) for our coral proteins in AlphaFold DB. By the aid of PyMOL, we selected 400 structures and aligned them to public AlphaFold structures of their similar proteins (BLAST E value < 2.8e-309) instead, then calculated RMSD. Most structure pairs seem to have little difference (Fig S3), but some do differ significantly. Considering the lack of exactly same coral proteins in AlphaFold DB, it might be difficult to detect whether the sequential feature or the AI system itself is responsible for those differences. It is proposed that until more AlphaFold-registered or experimentally verified structures are available, pLDDT will remain the main technical control indicator for coral protein structure prediction, meanwhile our resulting dataset may temporarily serve as a coral-specific extension of AlphaFold DB for RMSD analysis (coral biologists can search our dataset for the same proteins and calculate RMSD).

5. Response to comment: The data dump would be stronger if the mass spec proteomics data was also made available. What proportion of the structural proteome has mass-spectral support?
The mass spectrometry proteomics data have been deposited to the ProteomeXchange Consortium via the iProX partner repository with the dataset identifier PXD034973, and they will be made public once our manuscript is published. All the 8,382 proteins have mass-spectral support, and quantification of their expression level is available in Table S3.

6. Response to comment: Please include a supplementary text file containing the key features of each predicted protein.
We have provided Table S4 for all key features. We have also developed a browse page (http://corals.bmeonline.cn/prot/browse.php) for our web server.

7. Response to comment: The long proteins could be split by domain to provide some structural information.
This suggestion is excellent. For long proteins, AlphaFold Protein Structure Database just split them into 1400AA fragments with 200AA overlap. Splitting long proteins by domain before prediction should be more appropriate, yet there is no existing tool for this. Since predicting structures of long fragments of the remaining 216 proteins (933,369 residues, average length 4,321 AA) may be time-consuming while one of our authors need the publication of this paper for his PhD, we fail to add this work to our revised manuscript, but we will gradually add these partial structure predictions to our web server, and we will try to customize a pipeline following your suggestions and apply it into subsequent studies. Besides, we are negotiating with colleagues to borrow a Nvidia Tesla A100 (80G GPU memory) for directly structure prediction of some long proteins.

8. Response to comment: To boost the value of this data, the authors might also consider predicting the coral symbiont proteomes followed by integrative analysis of host and symbiont proteomes to predict interacting partners.
This work has been already planned. We have generated the multiple sequence alignments of more than 90,000 Symbiont proteins for structure prediction, and the Big Data Computing Center of Southeast University have promised to triple the number of available GPUs for us if our manuscript is accepted. Of course, we will prioritize the remaining 216 long proteins in this work once we receive added computing resources.

9. Response to comment: What are the domain and sequence features of the low and very-low confidence predictions?
They are “orphan sequences” with few homologous sequences. AI systems depending on coevolutionary information do not do well with these sequences.

10. Response to comment: Is the reference genome available for any species? How does the mass spec and structural data improve the genome annotation and vice versa?
Our team has assembled the genomes of investigated corals to the chromosome level, yet whole genome annotation is still a work in progress. From our experience, mass spectrometry technology enables researchers to obtain high-throughput protein information from relatively small samples in standardized steps, and structural data

Powered by Editorial Manager® and ProduXion Manager® from Aries Systems Corporation

	enable researchers to use computational biology methods (such as molecular docking, https://doi.org/10.3389/fphys.2021.759370) to find key proteins and explore crucial physiological functions, providing evidence more reliable than sequence alignment. It is particularly beneficial for studies on several non-model organisms represented by corals. These species may be important to our ecology and society, but existing experimental protocols for them are not as perfect as those for model organisms, moreover people tend to focus on their potentially valuable components rather than a head-to-tail understanding of them.<br><br>11. Response to comment: At present large parts of the discussion are irrelevant.<br>We apologize for our first author's overexcitement at the time. We have deleted irrelevant arguments regarding COVID 19 and the call to arms for bioinformaticians.<br><br>12. Response to comment: Comment on whether toxicity is reported for these coral species.<br>No comment on toxicity is reported for these coral species, yet several toxic substances were found in their relatives (Acropora digitifera, Gacesa R, 2015; Montipora capitata, Hagedorn M, 2015). From our experience, our investigated corals are unlikely to be as dangerous as Millepora corals.<br><br>We look forward to hearing from you regarding our submission, and we would be glad to respond to any further questions and comments that you may have.<br><br>Yours sincerely, on behalf of all the authors,<br><br>Chunpeng He<br>State Key Laboratory of Bioelectronics, School of Biological Science and Medical Engineering, Southeast University, Nanjing, China.<br>e-mail: cphe@seu.edu.cn<br><br>Zuhong Lu<br>State Key Laboratory of Bioelectronics, School of Biological Science and Medical Engineering, Southeast University, Nanjing, China.<br>e-mail: zhlu@seu.edu.cn
**Additional Information:**	
**Question**	**Response**
Are you submitting this manuscript to a special series or article collection?	No
**Experimental design and statistics**<br><br>Full details of the experimental design and statistical methods used should be given in the Methods section, as detailed in our Minimum Standards Reporting Checklist. Information essential to interpreting the data presented should be made available in the figure legends.<br><br>Have you included all the information requested in your manuscript?	Yes
**Resources**	Yes

A description of all resources used, including antibodies, cell lines, animals and software tools, with enough information to allow them to be uniquely identified, should be included in the Methods section. Authors are strongly encouraged to cite Research Resource Identifiers (RRIDs) for antibodies, model organisms and tools, where possible.<br><br>Have you included the information requested as detailed in our Minimum Standards Reporting Checklist?	
**Availability of data and materials**<br><br>All datasets and code on which the conclusions of the paper rely must be either included in your submission or deposited in publicly available repositories (where available and ethically appropriate), referencing such data using a unique identifier in the references and in the "Availability of Data and Materials" section of your manuscript.<br><br>Have you have met the above requirement as detailed in our Minimum Standards Reporting Checklist?	Yes

Manuscript Click here to access/download;Manuscript;manuscript_R1.docx

Utilizing artificial intelligence system to build the digital structural proteome of reef-building corals

Yunchi Zhu[1], Xin Liao[2], Tingyu Han[1], J.-Y. Chen[3], Chunpeng He[1*] and Zuhong Lu[1*]

[1] State Key Laboratory of Bioelectronics, Southeast University, Nanjing, Jiangsu, China
[2] Guangxi Key Lab of Mangrove Conservation and Utilization, Guangxi Mangrove Research Center, Beihai, Guangxi, China
[3] Nanjing Institute of Paleontology and Geology, Nanjing, China

* Corresponding author(s):
E-mail: cphe@seu.edu.cn; zhlu@seu.edu.cn

Abstract

Background Reef-building corals play an important role in the marine ecosystem, and analyzing their proteomes from a structural perspective will exert positive effects on exploring their biology. Here we integrated mass spectrometry with newly published ColabFold to obtain digital structural proteomes of dominant reef-building corals. **Results** 8,382 homologous proteins in *Acropora muricata*, *Montipora foliosa* and *Pocillopora verrucosa* were identified, then 8,166 of them got predicted structures after about 4,060 GPU hours of computation. The resulting dataset covers 83.6% of residues with a confident prediction, while 25.9% have very high confidence. **Conclusions** Our work provides insight-worthy predictions for coral research, confirms the reliability of ColabFold in practice, and is expected to be a reference case in the impending high-throughput era of structural proteomics.

Keywords

Reef-building coral; ColabFold; Structural proteomics

Introduction

Coral reefs serve as a living environment for more than 30% of marine animals and plants [1-2], while they are suffering from sharply declining due to global warming, changes in the physicochemical environment of the ocean, and massive encroachment of the predatory crown-of-thorns starfish [3-7]. Several researchers even propose that features similar to those exhibited during the last mass extinction have emerged in scleractinian coral populations, including population shrinkage, transplanting of colonies to the aphotic zone, and zygote dormancy [8-9]. Such serious situation has brought about research focused on the growth, restoration, and ecological defence of reef-building corals.

However, in contrast to more common model organisms, there remains a lack of public omics data from reef-building corals, no exception for the proteome bridging physiological function and genome. Taking the UniProt database as an example, as of June 2022, it has collected no more than 50 reviewed proteins from *Acropora*, one of the most species rich coral genera, while for another two dominant genera *Montipora* and *Pocillopora*, the number even fails to reach 5. Various factors such as geographic location and technical limitations result in the "data gap" for coral research [10], and to make matters worse, COVID-19 has exacerbated the risks of sampling at wild as well as performing "wet" experiments. Mass spectrometry technology having been applied in coral proteomics research [11-14] enables researchers to obtain high-throughput protein information from relatively small samples in standardized steps, yet according to traditional protocols, downstream analysis on protein structures and functions still requires cumbersome manual operations.

The booming AI (artificial intelligence) technology is expected to provide new solutions for current predicament. The results of the biennial Critical Assessment of protein Structure Prediction (CASP) have revealed the substantial progress in protein structure prediction [15]. In CASP14 (2020), AlphaFold2 [16] achieving a record score of 92.4 was brought to spotlight. As the structure modelling solution challenging x-ray crystallography and cryo-electron microscopy, AlphaFold2 can directly transform sequences to structures with high accuracy, particularly beneficial for studies on several non-model organisms represented by corals. These species may be important to our ecology and society, but existing experimental protocols for them are not as perfect as those for model organisms, moreover people tend to focus on their potentially valuable components rather than a head-to-tail understanding of them. Assisted by AI modelling, scientists can rapidly acquire their structural proteomes [17]

in the digital lab, then use computational biology methods to find key proteins and explore crucial physiological functions, so as to improve genome annotation and pave the way for applications including breeding and protection.

Compared to other prediction algorithms such as RoseTTAFold [18], AlphaFold2 has obvious disadvantages in performance [19], as even protein domains can consume long computation time [20]. Fortunately, its open source has attracted joint efforts of developers to improve it. In 2021, Zhong and colleagues from the center for high performance computing (HPC) of Shanghai Jiao Tong University released ParaFold [21], the specific AlphaFold version for their HPC clusters, making a successful attempt to accelerate AlphaFold2. In May 2022, ColabFold [22] claiming to make protein folding accessible to all got officially published. Replacing Jackhmmer with MMseqs2 [23] and utilizing optimized model, ColabFold is able to run dozens of times faster than original AlphaFold2, which means it greatly expands the throughput of protein structure modelling, thus breaking the last hurdle in effectively predicting complete structural proteome.

Here we integrated mass spectrometry with AI system to obtain digital structural proteomes of dominant reef-building corals. Deploying ColabFold on the Big Data Computing Center of Southeast University, we predicted structures of more than 8,000 homologous proteins among *Acropora muricata, Montipora foliosa* and *Pocillopora verrucosa* in approximately 4,060 GPU hours. The resulting dataset named CP-8382 covers 83.6% of residues with a confident prediction and 25.9% with very high confidence. We also developed a search engine interface in the style of AlphaFold Protein Structure Database [24] (AFDB for short) to open our data to the community (http://corals.bmeonline.cn/prot/).

Materials and Methods

Experimental model and subject details

The species including *A. muricata*, *M. foliosa* and *P. verrucosa* in the study were collected from the Xisha Islands in the South China Sea (latitude 15°40'–17°10' north, longitude 111°–113° east).

The coral samples were cultured in our laboratory coral tank with conditions conforming to their habitat environment. All the species were raised in a RedSea® tank (redsea575, Red Sea Aquatics Ltd) at 26°C and 1.025 salinity (Red Sea Aquatics Ltd). The physical conditions of the coral culture system are as follows: three coral lamps (AI®, Red Sea Aquatics Ltd), a protein skimmer (regal250s, Reef Octopus), a water chiller (tk1000, TECO Ltd), two wave devices (VorTech^{TM} MP40, EcoTech Marine Ltd), and a calcium reactor (Calreact 200, Reef Octopus), etc.

Total Protein Extraction

Sample was ground individually in liquid nitrogen and lysed with PASP lysis buffer (100 mM NH_4HCO_3, 8 M Urea, pH 8), followed by 5 min of ultrasonication on ice. The lysate was centrifuged at 12000 g for 15 min at 4°C and the supernatant was reduced with 10 mM DTT for 1h at 56°C, and subsequently alkylated with sufficient IAM for 1 h at room temperature in the dark. Then samples were completely mixed with 4 times volume of precooled acetone by vortexing and incubated at -20°C for at least 2h. Samples were then centrifuged at 12000 g for 15 min at 4°C and the precipitation was collected. After washing with 1mL cold acetone, the pellet was dissolved by dissolution buffer (8 M Urea, 100 mM TEAB, pH 8.5).

Protein Quality Test

BSA standard protein solution was prepared according to the instructions of Bradford protein quantitative kit, with gradient concentration ranged from 0 to 0.5 g/L. BSA standard protein solutions and sample solutions with different dilution multiples were added into 96-well plate to fill up the volume to 20 µL, respectively. Each gradient was repeated three times. The plate was added 180 µL G250 dye solution quickly and placed at room temperature for 5 minutes, the absorbance at 595 nm was detected. The standard curve was drawn with the absorbance of standard protein solution and the protein concentration of the sample was calculated. 20 µg of the protein sample was loaded to 12% SDS-PAGE gel electrophoresis, wherein the concentrated gel was performed at 80 V for 20 min, and the separation gel was performed at 120 V for 90 min. The gel was stained by coomassie brilliant blue R-250 and decolored until the bands were visualized clearly.

TMT Labeling of Peptides

Each protein sample was taken and the volume was made up to 100 µL with DB dissolution buffer (8 M Urea, 100 mM TEAB, pH 8.5). Trypsin and 100 mM TEAB buffer were added, sample was mixed and digested at 37 °C for 4h. And then, trypsin and $CaCl_2$ were added, sample was digested overnight. Formic acid was mixed with digested sample, adjusted pH under 3, and centrifuged at 12000 g for 5 min at room temperature. The supernatant was slowly loaded to the C18 desalting column, washed with washing buffer (0.1% formic acid, 3% acetonitrile) 3 times, then eluted by some elution buffer (0.1% formic acid, 70% acetonitrile). The eluents of each sample were collected and lyophilized. 100 µL of 0.1 M TEAB buffer was added to reconstitute, and 41 µL of acetonitrile-dissolved TMT labeling reagent was added, sample was mixed with shaking for 2 h at room temperature. Then, the reaction was stopped by adding 8% ammonia. All labeling samples were mixed with equal volume, desalted and lyophilized.

Separation of fractions

Mobile phase A (2% acetonitrile, adjusted pH to 10.0 using ammonium hydroxide) and B (98% acetonitrile) were used to develop a gradient elution. The lyophilized powder was dissolved in solution A and centrifuged at 12,000 g for 10 min at room temperature. The sample was fractionated using a C18 column (Waters BEH C18, 4.6×250 mm, 5 µm) on a Rigol L3000 HPLC system, the column oven was set as 45°C. The detail of elution gradient was shown in **Table S1**. The eluates were monitored at UV 214 nm, collected for a tube per minute and combined into 10 fractions finally. All fractions were dried under vacuum, and then, reconstituted in 0.1% (v/v) formic acid (FA) in water.

LC-MS/MS analysis

For transition library construction, shotgun proteomics analyses were performed using an EASY-nLC™ 1200 UHPLC system (Thermo Fisher) coupled with a Q Exactive™ series mass spectrometer (Thermo Fisher) operating in the data-dependent acquisition (DDA) mode. 1 µg sample was injected into a home-made C18 Nano-Trap column (4.5 cm×75 µm, 3 µm). Peptides were separated in a home-made analytical column (15 cm×150 µm, 1.9 µm), using a linear gradient elution as listed in **Table S2**. The separated peptides were analyzed by Q Exactive™ series mass spectrometer (Thermo Fisher), with ion source of Nanospray Flex™ (ESI), spray voltage of 2.3 kV and ion transport capillary temperature of 320°C. Full scan ranges from m/z 350 to 1500 with resolution of 60000 (at m/z 200), an automatic gain control (AGC)

target value was 3×10^6 and a maximum ion injection time was 20 ms. The top 40 precursors of the highest abundant in the full scan were selected and fragmented by higher energy collisional dissociation (HCD) and analyzed in MS/MS, where resolution was 45000 (at m/z 200) for 10 plex, the automatic gain control (AGC) target value was 5×10^4 the maximum ion injection time was 86 ms, a normalized collision energy was set as 32%, an intensity threshold was 1.2×10^5, and the dynamic exclusion parameter was 20 s.

Protein identification and quantitation

The resulting spectra from each run were searched separately against protein-coding sequences from NCBI Bioproject PRJNA544778 by Proteome Discoverer 2.2 (PD 2.2, Thermo) [25]. The searched parameters are set as follows: mass tolerance for precursor ion was 10 ppm and mass tolerance for product ion was 0.02 Da. Carbamidomethyl was specified as fixed modifications, Oxidation of methionine (M) and TMT plex were specified as dynamic modification, acetylation and TMT plex were specified as N-Terminal modification in PD 2.2. A maximum of 2 miscleavage sites were allowed.

In order to improve the quality of analysis results, the software PD 2.2 further filtered the retrieval results: Peptide Spectrum Matches (PSMs) with a credibility of more than 99% were identified PSMs. The identified protein contains at least 1 unique peptide. The identified PSMs and protein were retained and performed with FDR no more than 1.0%.

Structure modelling

Protein sequences were sorted by length and numbered as CPXXXXXXXX according to order before sent to ColabFold (1.3.0) platform. 1,053 proteins no longer than 200 aa were calculated on NVIDIA Tesla P100 while others were calculated on NVIDIA Tesla V100 cluster of the Big Data Computing Center of Southeast University. The parameters of ColabFold were set to *--amber*, *--templates*, *--num-recycle 3*, *--use-gpu-relax*. For each protein, structure with highest pLDDT scores (**_relaxed_rank_1_model_x.pdb*) was preserved and labeled as CPXXXXXXXX.pdb.

400 structures predicted in this work were selected and aligned to public AlphaFold structures of their similar proteins (BLAST E value < 2.8e-309) by PyMOL, then RMSDs were calculated.

Protein annotation

Each coral protein was annotated by NR (diamond v2.0.14.152, *blastp --evalue 1e-5 -k 1*), UniProt (same tool and parameter as NR) and InterPro (interproscan-5.54-87.0). Pfam annotations for them were got via eggNOG-mapper [26].

Template search results generated by ColabFold (**.template_domain_names.json*) were compared to SCOP2 [27] and CATH [28].

Search engine development

Elasticsearch 7.12.1 was employed as the key module, where NR, UniProt and InterPro annotations were transformed into keywords in index. Web interface was implemented using PHP 7.2, while Nginx worked as web server.

Sequenceserver [29] 2.0.0 were deployed as the BLAST server, source codes of which were modified to link each hit to its information page. Mol* [30] in the style of AFDB was imported as structure viewer.

Results and Discussion

Protein identification and annotation

8,382 homologous proteins in *A. muricata*, *M. foliosa* and *P. verrucosa* were identified by Proteome Discoverer, sequences and expression profile of which are shown in **Table S3**. It is assumed that a considerable portion of proteomes be conserved among these dominant coral genera, for the total protein-coding gene number of one scleractinian coral is unlikely to exceed 25,000 according to previous reports [9-10][31]. Over 97% of them are shorter than 2,600 aa, while the longest is up to 14,622 aa.

Proteins were annotated with NR and UniProt for homologs, and InterPro for domains. NR annotations map most proteins to scleractinians (**Fig 1.A**), rather than other cnidarians or marine organisms, with an average identity beyond 90% (**Fig 1.B**). It indicates the efforts into filling the "data gap" hindering coral research [10], as an increasing number of coral sequences have been added into public databases in recent years. Although according to **Fig 1.B**, coral sequences in UniProt may not be as abundant as NR, the two databases could complement each other to improve annotation. **Fig 1.C** demonstrates domains frequently found on coral proteins. EF-hand domain related to calcium signalling pathways [32] gets the top 1, just consistent with these corals' roles as reef builders, who deal with calcium ion every day for skeleton construction and homeostatic regulation. The RNA recognition motif domain and protein kinase domain present to be common protein domains in eukaryotes. Von Willebrand factor (type A) may play an important role in coral immunity, as it has been reported to participate in allorejection responses [33]. WD40 repeat with "doughnut hole" usually serve as protein interaction scaffolds in multiprotein complexes [34], which might also regulate innate immunity and stress response [35-36].

Additionally, Pfam annotation of the coral proteome was made as a supplement to InterPro, and statistics of Pfam families are shown in **Fig S1**. All annotations are available in **Table S4**.

Protein structure prediction

After about 4,060 GPU hours of computation, ColabFold succeeded to generate 8,166 structures, covering 97.4% of our coral protein dataset and touching the maximum length that an NVIDIA Tesla V100 (32 GB) can process. The model confidence distribution is presented in **Fig 2**. In resulting dataset 83.6% of residues have pLDDT (predicted local distance difference test) larger than 70, which are considered as confident predictions [15], and 25.9% get very high pLDDT over 90. No more than 2% of predicted structures have an average pLDDT below 50. The predictions from ColabFold can be recognized as credible overall.

An attempt was made to compare the predicted structure models to public protein structural classification database. ColabFold totally found 27,887 template domains, among which nearly 70% have been registered in SCOP or CATH (**Fig S2**). Nevertheless, current template search results turn out to be sequence-based, not final-structure-based, thus using them to represent fold distributions might fail to avoid false positives. It would be better to apply high-throughput structure alignment methods into classification of remaining template domains as well as annotation of various novel folds, yet existing methods are not efficient enough to handle the increasing scale of data pushed from AI systems.

Differences between AlphaFold and ColabFold predictions were also observed. 400 structures in the resulting dataset were aligned to public AlphaFold structures of their similar proteins, then RMSDs were calculated. As illustrated in **Fig S3**, most structure pairs seem to have little difference, but some do differ significantly. Considering the lack of exactly same coral proteins in AFDB, it might be difficult to detect whether the sequential feature or the AI system itself is responsible for those differences. Hence, until more AlphaFold-registered or experimentally verified structures are available, pLDDT will remain the main technical control indicator for coral protein structure prediction, meanwhile the resulting dataset of this work may be able to temporarily serve as a coral-specific extension of AFDB for RMSD analysis.

Highlighted predictions

Fig 3 demonstrates several highlighted structure predictions from the resulting dataset. There are few doubts that biomineralization is the most significant function of reef-building corals [37-38], thus their skeletal proteome regulating the mineral deposition is always of interest to scientists. Our results cover most of recognized coral mineralization-related proteins, including skeletal organic matrix protein (SOMP), skeletal aspartic acid-rich protein, collagen, carbonic anhydrase, etc. [11-12] **Fig 3.A** shows the sequence and structure alignment among three acidic SOMPs, two identified in this work and one from UniProt (B3EWY7). Although the sequence identity is only 40%-60%, it appears that their structures be broadly similar, consisting of an Asp-rich tongue-like region and a β-sheet-formed region. According to previous studies, Asp-rich proteins are supposed to interact directly with calcium carbonate crystals promoting crystal nucleation, determining the growth axes and inhibiting the crystal growth [39-40], and they usually have high-capacity yet low-affinity calcium-binding properties [41]. Our structure predictions may provide an explanation that for these proteins Asps are concentrated in an open tongue-like region, reducing the steric hindrance while weakening the binding strength with calcium ions. Besides, the β-sheet-rich region at the base of the "Asp tongue" form a barrel-like local structure, which is also found in many other uncharacterized skeletal organic matrix proteins (USOMP) (**Fig S4**). These barrel-like regions probably have transmembrane functions [42], however existing methods fail to annotate them with any known domains, making these SOMPs uncharacterized. This phenomenon might not only urge biologists to gain deeper insights into protein domains, but also enlighten bioinformaticians to design novel annotation algorithms for 3D structure data.

Toxins are widely employed by marine organisms for prey capture and defense of the territory [43]. Natterin proteins discovered in the venom of the medically significant Brazilian toadfish *Thalassophryne nattereri* [44] are representative perforin-like toxins which can insert into the lipid bilayer to trigger the disruption of membrane function [45]. We identify one Natterin-4-like protein CP00002607, as illustrated in **Fig 3.B**. The drill-like toxic domain is supposed to "bore holes" on membranes, resulting in electrolyte leakage and inflammation [46]. The function of DUF3421 has not been commonly recognized [47], though several researchers propose such DM9-containing proteins be new members of PRRs (pattern recognition receptors) [48-50]. From a structural point of view, this DM9-containing domain is at the end of "toxic drill" with a funnel-like cavity. It indeed has potential to bind signalling molecule playing roles in the antagonism between toxin and immune system, nevertheless the possibility that it just exacerbates membrane damage or attaches other toxic chemicals cannot be ruled out. This structure prediction might help to deepen our understanding of marine biotoxin and provide inspiration for drug development [51], not to mention that it can serve as raw input for molecular docking itself.

Symbiosis is another important topic for coral biologists and ecologists, as reef-building corals obtain the majority of their energy and nutrients from their algal symbionts (mainly

Symbiodiniaceae [52]), and loss of symbionts is causing coral bleaching threatening marine ecology. Improved knowledge of interpartner signalling in coral holobionts could be applied to solutions against the coral reef crisis [53]. Previous studies reveal that the initiation of coral symbiosis depends on the interaction between PRRs on host gastrodermal nutritive phagocytes and MAMPs (microbe-associated molecular patterns) of Symbiont [54-55], as shown in **Fig 3.C**. Complete structures of several representative PRRs, such as C3R (complement 3 receptor), TLR (toll-like receptor), lectin and SR (scavenger receptor), get confident prediction in our work with pLDDT ranging from 75 to 88. Unfortunately, our predictions fail to cover PRRs too large for our GPU devices to process, and symbiont proteins including MAMPs are beyond the scope of our experiment design. It will be an excellent work to acquire Symbiodiniaceae structural proteomes and integrate them with corals', which may bring about novel insights into coral symbiosis as well as interpartner signalling in cnidarians. In fact, the "data gap" for symbionts in coral holobionts turns out to be even larger than that for corals themselves [10], presenting both challenges and opportunities.

Web interface of CP-8382

Combining annotations and predicted structures, the resulting dataset was named as CP-8382 and curated into an online database (http://corals.bmeonline.cn/prot/). **Fig 4** describes its data content as well as the workflow of corresponding web interface. Predicted structures are displayed in the style of AFDB, where pLDDT of each residue is marked by color, and Mol* app enables users to zoom, rotate or take screenshot. They can be downloaded in the format of PDB or CIF. PAE (Predicted aligned error) graphs are given for assessing confidence in global features [15], raw data of which can be accessed in JSON format. Annotations with keywords highlighted are also presented. All the above information is accessible via search engine or BLAST server at the web interface. **Video S1** provides a demo of searching skeletal aspartic acid-rich protein in CP-8382.

Conclusion

By the aid of ColabFold, we succeeded to generate a content-rich structure dataset for reef-building corals within an acceptable period. Its claim to make protein folding accessible to all is seemingly not an exaggeration, furthermore the relatively high confidence of our results might prove that ColabFold does not sacrifice too much accuracy for speed [22].

Our work is expected to be an early case of digital structural proteome building, not just a contribution to coral research. Computational biologists preferring similar approaches may optimize their resource allocation referred to our experience. Moreover, moving most preliminary work to "dry lab" will not only accelerate research progress via HPC technology, but also help to break regional restrictions and boost brainstorming through the internet, so as to facilitate problem-solving in a more efficient and economical way. In view that the open source of RoseTTAFold and AlphaFold2 has brought the dawn of the high-throughput era of structural proteomics, we recommend researchers, especially those interested in unfamous yet potentially important organisms, to build and publish digital structural proteomes, which is conducive to new solutions and questions from a structural perspective, people's deeper understanding of biodiversity, and attraction of joint efforts.

Declarations

Ethics approval and consent to participate

All coral samples were collected and processed in accordance with local laws for invertebrate protection and approved by the Ethics Committee of Institutional Animal Care and Use Committee of Nanjing Medical University (protocol code IACUC-1910003 and date of approval is 10 October 2019).

Consent for publication

Not applicable.

Availability of data and materials

The mass spectrometry proteomics data have been deposited to the ProteomeXchange Consortium via the iProX partner repository [56] with the dataset identifier PXD034973.

CP-8382 dataset including all sequences, annotations and structure predictions is available at https://doi.org/10.6084/m9.figshare.20128265.v1, and we sincerely recommend acquiring data via our online database introduced in this paper (http://corals.bmeonline.cn/prot/).

Competing interests

The authors declare that they have no competing interests.

Funding

This work was supported by the open research fund of State Key Laboratory of Bioelectronics, Southeast University [Sklb2021-k02], and the open research fund program of Guangxi Key Lab of Mangrove Conservation and Utilization [GKLMC-202002].

Authors' contributions

YZ: experiment, database, writing and editing. TH: data uploading. ZL & JC: reviewing. CH: supervision. XL: project approval. All authors contributed to the article and approved the submitted version.

Acknowledgements

We are grateful for Dr. Xiaojun Xia’s technical support in our production environment.

We thank the Big Data Computing Center of Southeast University for providing the facility support on the numerical calculations in this paper.

References

1. Odum HT, Odum EP. Trophic structure and productivity of a windward coral reef community on Eniwetok Atoll. Ecological Monographs. 1955;25(3):291-320. doi:10.2307/1943285
2. Yu KF. Coral reefs in the South China Sea: Their response to and records on past environmental changes. Science China Earth Sciences. 2012;55(8):1217-1229. doi:10.1007/s11430-012-4449-5
3. Moberg F, Folke C. Ecological goods and services of coral reef ecosystems. Ecological Economics. 1999;29(2):215-233. doi:10.1016/S0921-8009(99)00009-9
4. WILSON SK, GRAHAM NA, PRATCHETT MS, JONES GP, POLUNIN NV. Multiple disturbances and the global degradation of coral reefs: are reef fishes at risk or resilient? GLOBAL CHANGE BIOLOGY. 2006;12(11):2220-2234. doi: 10.1111/j.1365-2486.2006.01252.x
5. Nakamura M, Okaji K, Higa Y, Yamakawa E, Mitarai S. Spatial and temporal population dynamics of the crown-of-thorns starfish, Acanthaster planci, over a 24-year period along the central west coast of Okinawa Island, Japan. Marine Biology. 2014;161(11):2521-2530. doi:10.1007/s00227-014-2524-5
6. Reimer JD, Kise H, Wee HB, Lee C-L, Soong K. Crown-of-thorns starfish outbreak at oceanic Dongsha Atoll in the northern South China Sea. Marine Biodiversity. 2019;49(6):2495-2497. doi:10.1007/s12526-019-01021-2
7. Magel Jennifer M. T., Dimoff Sean A., Baum Julia K. Direct and Indirect Effects of Climate Change-Amplified Pulse Heat Stress Events on Coral Reef Fish Communities. Bulletin of the Ecological Society of America. 2020;101(3):1-6. Accessed February 9, 2022. doi: 10.1002/bes2.1706
8. Dishon G, Grossowicz M, Krom M, Guy G, Gruber DF, Tchernov D. Evolutionary Traits that Enable Scleractinian Corals to Survive Mass Extinction Events. Sci Rep. 2020;10(1):3903. Published 2020 Mar 3. doi:10.1038/s41598-020-60605-2
9. Guo Z, Liao X, Chen JY, He C, Lu Z. Binding Pattern Reconstructions of FGF-FGFR Budding-Inducing Signaling in Reef-Building Corals. Front Physiol. 2022;12:759370. Published 2022 Jan 4. doi:10.3389/fphys.2021.759370
10. Zhu Y, Liao X, Han T, Chen JY, He C, Lu Z. Symbiodiniaceae microRNAs and their targeting sites in coral holobionts: A transcriptomics-based exploration. Genomics. 2022;114(4):110404. doi:10.1016/j.ygeno.2022.110404
11. Ramos-Silva P, Kaandorp J, Huisman L, et al. The skeletal proteome of the coral Acropora millepora: the evolution of calcification by co-option and domain shuffling. Mol Biol Evol. 2013;30(9):2099-2112. doi:10.1093/molbev/mst109
12. Drake JL, Mass T, Haramaty L, Zelzion E, Bhattacharya D, Falkowski PG. Proteomic analysis of skeletal organic matrix from the stony coral Stylophora pistillata. Proc Natl Acad Sci U S A. 2013;110(10):3788-3793. doi:10.1073/pnas.1301419110
13. Conci N, Lehmann M, Vargas S, Wörheide G. Comparative Proteomics of Octocoral and Scleractinian Skeletomes and the Evolution of Coral Calcification. Genome Biol Evol. 2020;12(9):1623-1635. doi:10.1093/gbe/evaa162
14. Peled Y, Drake JL, Malik A, et al. Optimization of skeletal protein preparation for LC-MS/MS sequencing yields additional coral skeletal proteins in Stylophora pistillata. BMC Mater. 2020;2:8. Published 2020 Jul 16. doi:10.1186/s42833-020-00014-x
15. Tunyasuvunakool K, Adler J, Wu Z, et al. Highly accurate protein structure prediction for the human proteome. Nature. 2021;596(7873):590-596. doi:10.1038/s41586-021-03828-1
16. Jumper J, Evans R, Pritzel A, et al. Highly accurate protein structure prediction with AlphaFold. Nature. 2021;596(7873):583-589. doi:10.1038/s41586-021-03819-2

17. Yee A, Pardee K, Christendat D, Savchenko A, Edwards AM, Arrowsmith CH. Structural proteomics: toward high-throughput structural biology as a tool in functional genomics. Acc Chem Res. 2003;36(3):183-189. doi:10.1021/ar010126g
18. Baek M, DiMaio F, Anishchenko I, et al. Accurate prediction of protein structures and interactions using a three-track neural network. Science. 2021;373(6557):871-876. doi:10.1126/science.abj8754
19. Shenggan Cheng, Ruidong Wu, Zhongming Yu, et al. FastFold: Reducing AlphaFold Training Time from 11 Days to 67 Hours. CoRR. 2022;abs/2203.00854. doi:10.48550/arXiv.2203.00854
20. Zhu Y, Lu N, Chen JY, He C, Huang Z, Lu Z. Deep whole-genome resequencing sheds light on the distribution and effect of amphioxus SNPs. BMC Genom Data. 2022;23(1):26. Published 2022 Apr 8. doi:10.1186/s12863-022-01038-w
21. Zhong B, Su X, Wen M, Zuo S, Hong L, Lin J. ParaFold: Paralleling AlphaFold for Large-Scale Predictions. International Conference on High Performance Computing in Asia-Pacific Region Workshops. January 2022:1-9. doi:10.1145/3503470.3503471
22. Mirdita M, Schütze K, Moriwaki Y, Heo L, Ovchinnikov S, Steinegger M. ColabFold: making protein folding accessible to all. Nat Methods. 2022;19(6):679-682. doi:10.1038/s41592-022-01488-1
23. Mirdita M, Steinegger M, Söding J. MMseqs2 desktop and local web server app for fast, interactive sequence searches. Bioinformatics. 2019;35(16):2856-2858. doi:10.1093/bioinformatics/bty1057
24. David A, Islam S, Tankhilevich E, Sternberg MJE. The AlphaFold Database of Protein Structures: A Biologist's Guide. J Mol Biol. 2022;434(2):167336. doi:10.1016/j.jmb.2021.167336
25. Orsburn BC. Proteome Discoverer-A Community Enhanced Data Processing Suite for Protein Informatics. Proteomes. 2021;9(1):15. Published 2021 Mar 23. doi:10.3390/proteomes9010015
26. Cantalapiedra CP, Hernández-Plaza A, Letunic I, Bork P, Huerta-Cepas J. eggNOG-mapper v2: Functional Annotation, Orthology Assignments, and Domain Prediction at the Metagenomic Scale. Mol Biol Evol. 2021;38(12):5825-5829. doi:10.1093/molbev/msab293
27. Andreeva A, Kulesha E, Gough J, Murzin AG. The SCOP database in 2020: expanded classification of representative family and superfamily domains of known protein structures. Nucleic Acids Res. 2020;48(D1):D376-D382. doi:10.1093/nar/gkz1064
28. Sillitoe I, Bordin N, Dawson N, et al. CATH: increased structural coverage of functional space. Nucleic Acids Res. 2021;49(D1):D266-D273. doi:10.1093/nar/gkaa1079
29. Priyam A, Woodcroft BJ, Rai V, et al. Sequenceserver: A Modern Graphical User Interface for Custom BLAST Databases. Mol Biol Evol. 2019;36(12):2922-2924. doi:10.1093/molbev/msz185
30. Sehnal D, Bittrich S, Deshpande M, et al. Mol* Viewer: modern web app for 3D visualization and analysis of large biomolecular structures. Nucleic Acids Res. 2021;49(W1):W431-W437. doi:10.1093/nar/gkab314
31. Shinzato C, Khalturin K, Inoue J, et al. Eighteen Coral Genomes Reveal the Evolutionary Origin of Acropora Strategies to Accommodate Environmental Changes. Mol Biol Evol. 2021;38(1):16-30.
32. Nelson MR, Thulin E, Fagan PA, Forsén S, Chazin WJ. The EF-hand domain: a globally cooperative structural unit. Protein Sci. 2002;11(2):198-205. doi:10.1110/ps.33302
33. Oren M, Amar KO, Douek J, Rosenzweig T, Paz G, Rinkevich B. Assembled catalog of immune-related genes from allogeneic challenged corals that unveils the participation of vWF-like transcript. Dev Comp Immunol. 2010;34(6):630-637. doi:10.1016/j.dci.2010.01.007

34. Schapira M, Tyers M, Torrent M, Arrowsmith CH. WD40 repeat domain proteins: a novel target class?. Nat Rev Drug Discov. 2017;16(11):773-786. doi:10.1038/nrd.2017.179
35. Kong D, Li M, Dong Z, Ji H, Li X. Identification of TaWD40D, a wheat WD40 repeat-containing protein that is associated with plant tolerance to abiotic stresses. Plant Cell Rep. 2015;34(3):395-410. doi:10.1007/s00299-014-1717-1
36. Liu WC, Li YH, Yuan HM, Zhang BL, Zhai S, Lu YT. WD40-REPEAT 5a functions in drought stress tolerance by regulating nitric oxide accumulation in Arabidopsis. Plant Cell Environ. 2017;40(4):543-552. doi:10.1111/pce.12723
37. Wang X, Zoccola D, Liew YJ, et al. The Evolution of Calcification in Reef-Building Corals. Mol Biol Evol. 2021;38(9):3543-3555. doi:10.1093/molbev/msab103
38. Von Euw S, Zhang Q, Manichev V, et al. Biological control of aragonite formation in stony corals. Science. 2017;356(6341):933-938. doi:10.1126/science.aam6371
39. Wheeler AP, George JW, Evans CA. Control of calcium carbonate nucleation and crystal growth by soluble matrx of oyster shell. Science. 1981;212(4501):1397-1398. doi:10.1126/science.212.4501.1397
40. Addadi L, Moradian J, Shay E, Maroudas NG, Weiner S. A chemical model for the cooperation of sulfates and carboxylates in calcite crystal nucleation: Relevance to biomineralization. Proc Natl Acad Sci U S A. 1987;84(9):2732-2736. doi:10.1073/pnas.84.9.2732
41. Maurer P, Hohenester E, Engel J. Extracellular calcium-binding proteins. Curr Opin Cell Biol. 1996;8(5):609-617. doi:10.1016/s0955-0674(96)80101-3
42. Fairman JW, Noinaj N, Buchanan SK. The structural biology of β-barrel membrane proteins: a summary of recent reports. Curr Opin Struct Biol. 2011;21(4):523-531. doi:10.1016/j.sbi.2011.05.005
43. Lima C, Disner GR, Falcão MAP, et al. The Natterin Proteins Diversity: A Review on Phylogeny, Structure, and Immune Function. Toxins (Basel). 2021;13(8):538. Published 2021 Jul 31. doi:10.3390/toxins13080538
44. Magalhães GS, Junqueira-de-Azevedo IL, Lopes-Ferreira M, Lorenzini DM, Ho PL, Moura-da-Silva AM. Transcriptome analysis of expressed sequence tags from the venom glands of the fish Thalassophryne nattereri. Biochimie. 2006;88(6):693-699. doi:10.1016/j.biochi.2005.12.008
45. Dal Peraro M, van der Goot FG. Pore-forming toxins: ancient, but never really out of fashion. Nat Rev Microbiol. 2016;14(2):77-92. doi:10.1038/nrmicro.2015.3
46. Greaney AJ, Leppla SH, Moayeri M. Bacterial Exotoxins and the Inflammasome. Front Immunol. 2015;6:570. Published 2015 Nov 10. doi:10.3389/fimmu.2015.00570
47. Ponting CP, Mott R, Bork P, Copley RR. Novel protein domains and repeats in Drosophila melanogaster: insights into structure, function, and evolution. Genome Res. 2001;11(12):1996-2008. doi:10.1101/gr.198701
48. Unno H, Matsuyama K, Tsuji Y, et al. Identification, Characterization, and X-ray Crystallographic Analysis of a Novel Type of Mannose-Specific Lectin CGL1 from the Pacific Oyster Crassostrea gigas. Sci Rep. 2016;6:29135. Published 2016 Jul 5. doi:10.1038/srep29135
49. Jiang S, Wang L, Huang M, et al. DM9 Domain Containing Protein Functions As a Pattern Recognition Receptor with Broad Microbial Recognition Spectrum. Front Immunol. 2017;8:1607. Published 2017 Nov 29. doi:10.3389/fimmu.2017.01607
50. Wang W, Song X, Wang L, Song L. Pathogen-Derived Carbohydrate Recognition in Molluscs Immune Defense. Int J Mol Sci. 2018;19(3):721. Published 2018 Mar 3. doi:10.3390/ijms19030721
51. Li Y, Orange JS. Degranulation enhances presynaptic membrane packing, which protects NK cells from perforin-mediated autolysis. PLoS Biol. 2021;19(8):e3001328. Published 2021 Aug 3. doi:10.1371/journal.pbio.3001328

52. LaJeunesse TC, Parkinson JE, Gabrielson PW, et al. Systematic Revision of Symbiodiniaceae Highlights the Antiquity and Diversity of Coral Endosymbionts. Curr Biol. 2018;28(16):2570-2580.e6. doi:10.1016/j.cub.2018.07.008
53. Rosset SL, Oakley CA, Ferrier-Pagès C, Suggett DJ, Weis VM, Davy SK. The Molecular Language of the Cnidarian-Dinoflagellate Symbiosis. Trends Microbiol. 2021;29(4):320-333. doi:10.1016/j.tim.2020.08.005
54. Davy SK, Allemand D, Weis VM. Cell biology of cnidarian-dinoflagellate symbiosis. Microbiol Mol Biol Rev. 2012;76(2):229-261. doi:10.1128/MMBR.05014-11
55. Weis VM. Cell Biology of Coral Symbiosis: Foundational Study Can Inform Solutions to the Coral Reef Crisis. Integr Comp Biol. 2019;59(4):845-855. doi:10.1093/icb/icz067
56. Ma J, Chen T, Wu S, et al. iProX: an integrated proteome resource. Nucleic Acids Res. 2019;47(D1):D1211-D1217. doi:10.1093/nar/gky869

Figures

Fig 1. Statistics of coral protein annotations. A. Homolog annotation with NR database. Horizontal axis is species and vertical axis is protein number. The top five species with the highest number of annotated proteins are *A. millepora*, *P. damicornis*, *A. digitifera*, *S. pistillata* and *O. faveolata*. Only 15 proteins fail to get any NR annotations. **B. Violin plot of identity distribution.** Horizontal axis is homolog database and vertical axis is identity. **C. Domain annotation with InterPro.** Horizontal axis is protein number and vertical axis is domain name. Top 20 domains with largest counts are displayed.

Fig 2. Distribution of model confidence against protein length. Horizontal axis is protein length and vertical axis is protein number. Model confidence calculated by pLDDT are color-coded. Very high: pLDDT > 90; Confident: 90 > pLDDT > 70; Low: 70 > pLDDT > 50; Very low: pLDDT < 50.

Fig 3. Highlighted structure predictions. A) Sequence and structure alignment among CP00002736, CP00003090 and B3EWY7. Asp-rich regions were framed in yellow and β-sheet-formed regions were framed in red. **B)** Structure of CP00002607 similar to natterin-4. **C)** Structures of several pattern recognition receptors involved in host-symbiont signalling, including C3R (complement 3 receptor), TLR (toll-like receptor), lectin and SR (scavenger receptor).

Fig 4. Content of CP-8382 dataset and workflow of its web interface. Users can utilize search engine or BLAST server to acquire their interested coral proteins on the website, where elasticsearch module will process inputs and return the following information: (I) Predicted structure displayed in the style of AFDB; (II) PAE graph, where the colour at position (x, y) indicates AlphaFold's expected position error at residue x when the predicted and true structures are aligned on residue y; (III) Annotations with keywords highlighted.

Fig 1

Click here to access/download;Figure;Fig_1.pdf

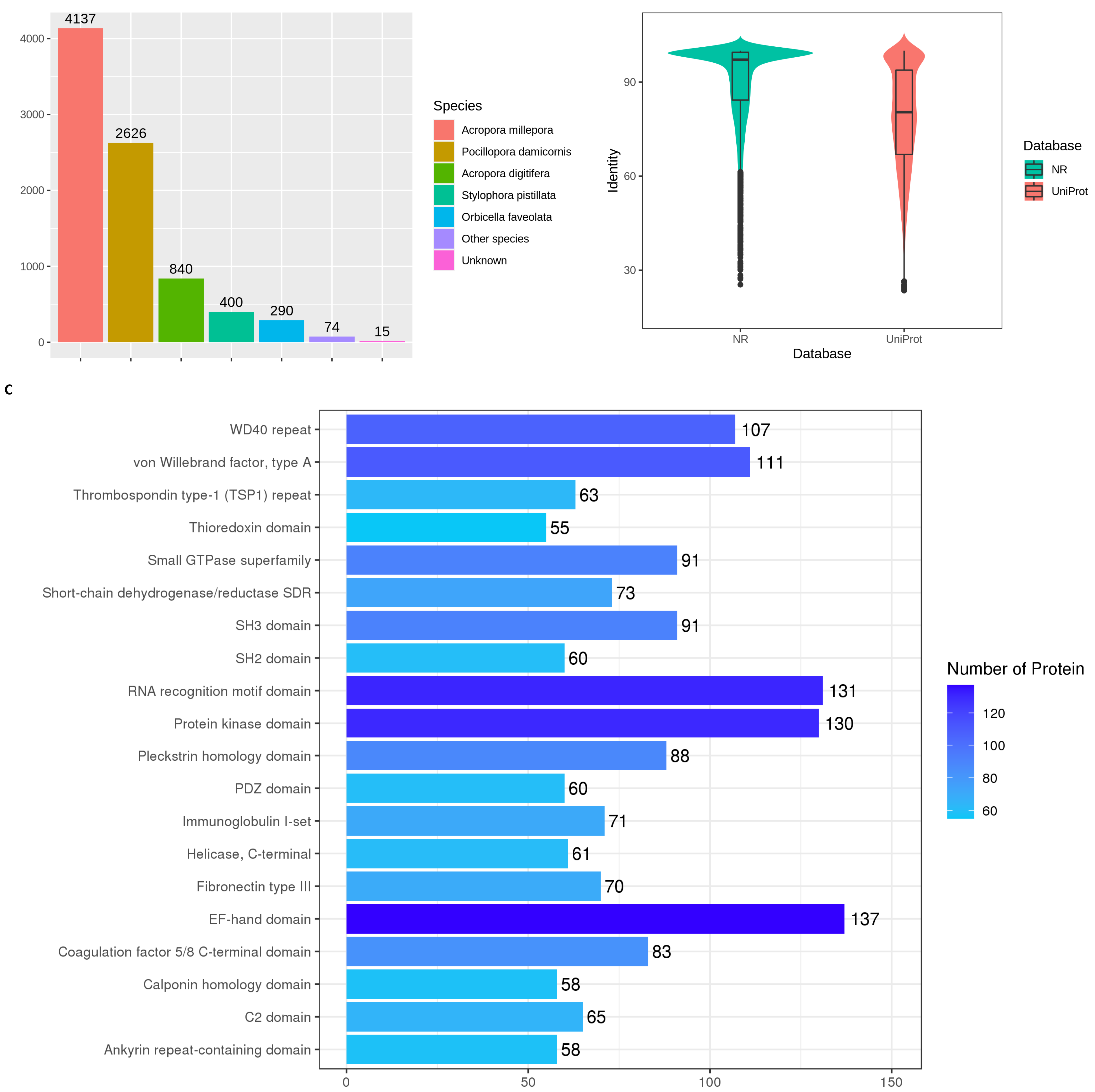

Click here to access/download;Figure;Fig_2.pdf

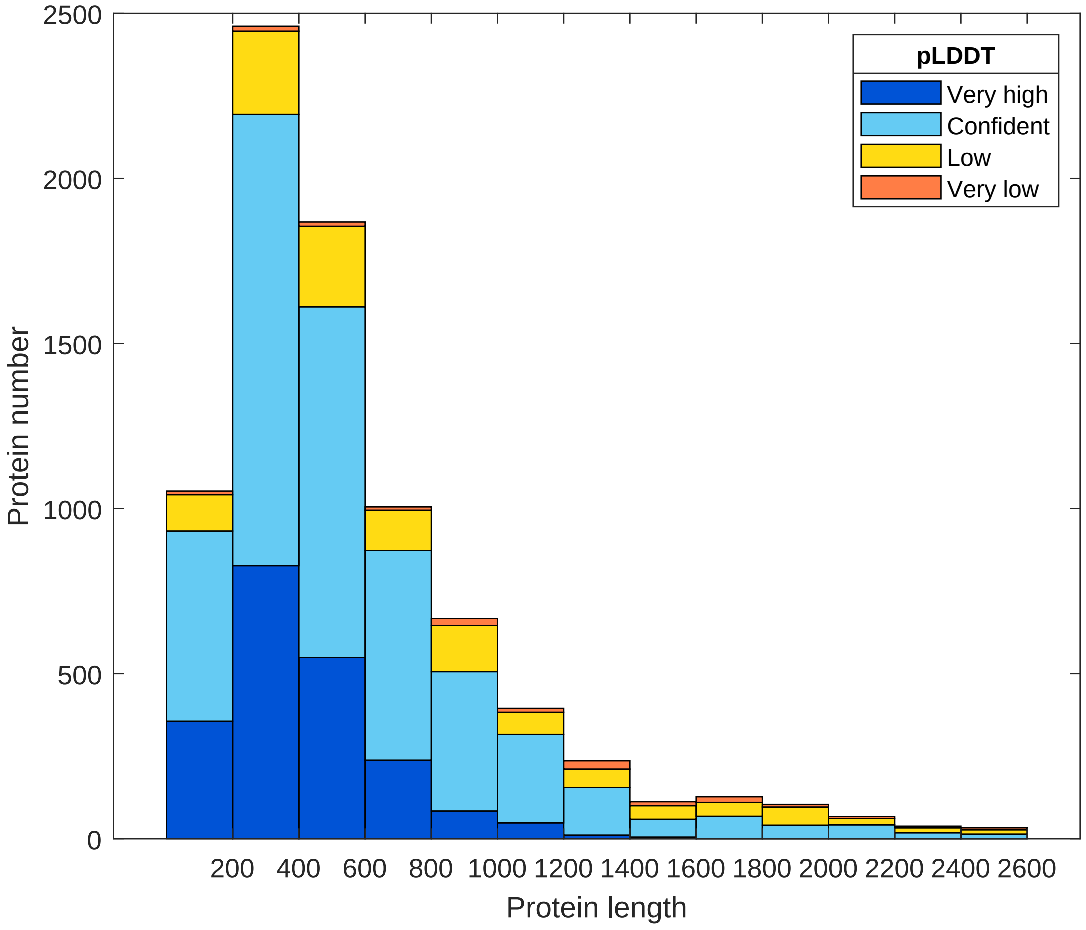

Click here to access/download;Figure;Fig_3.pdf

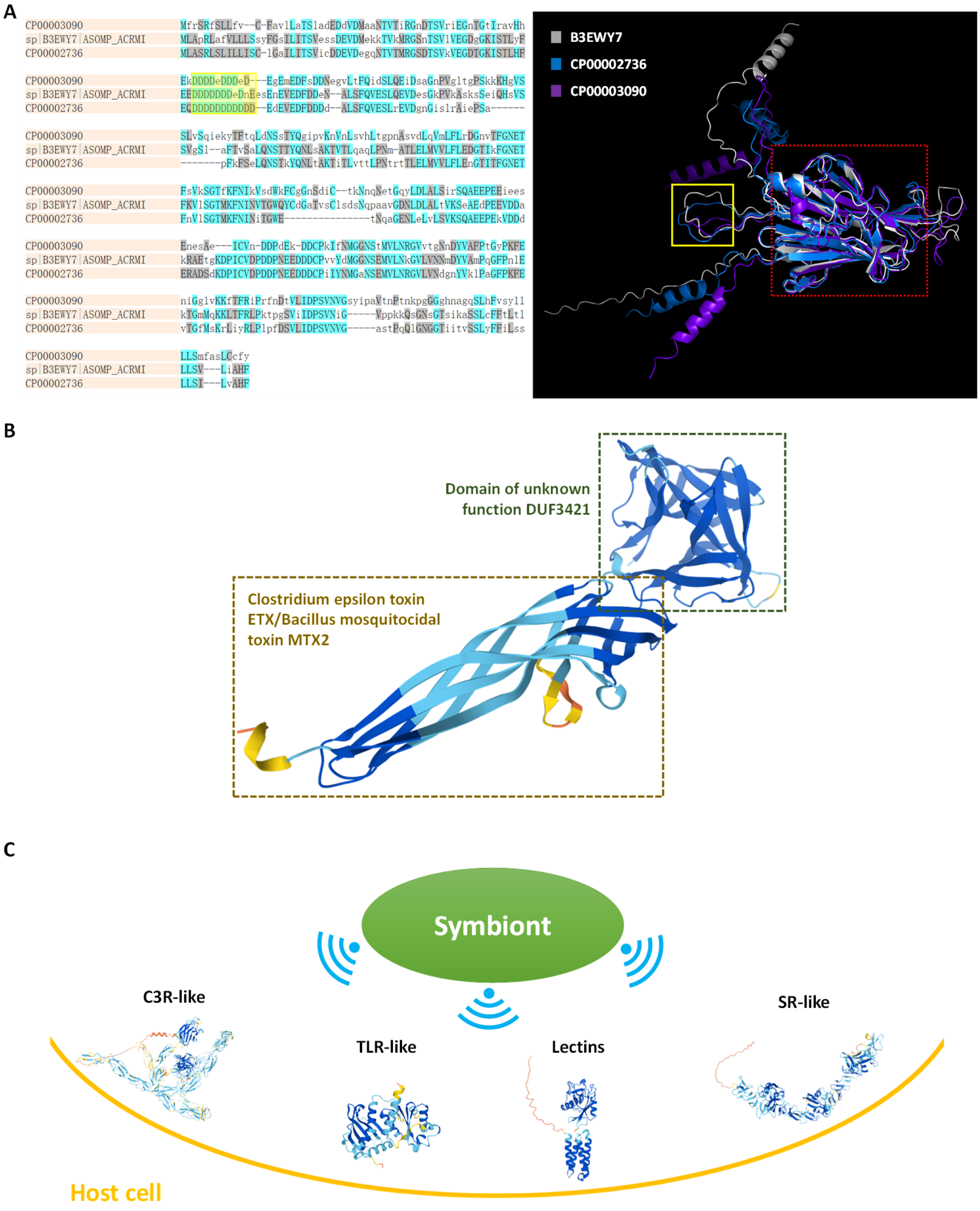

Fig 4

Click here to access/download;Figure;Fig_4.pdf

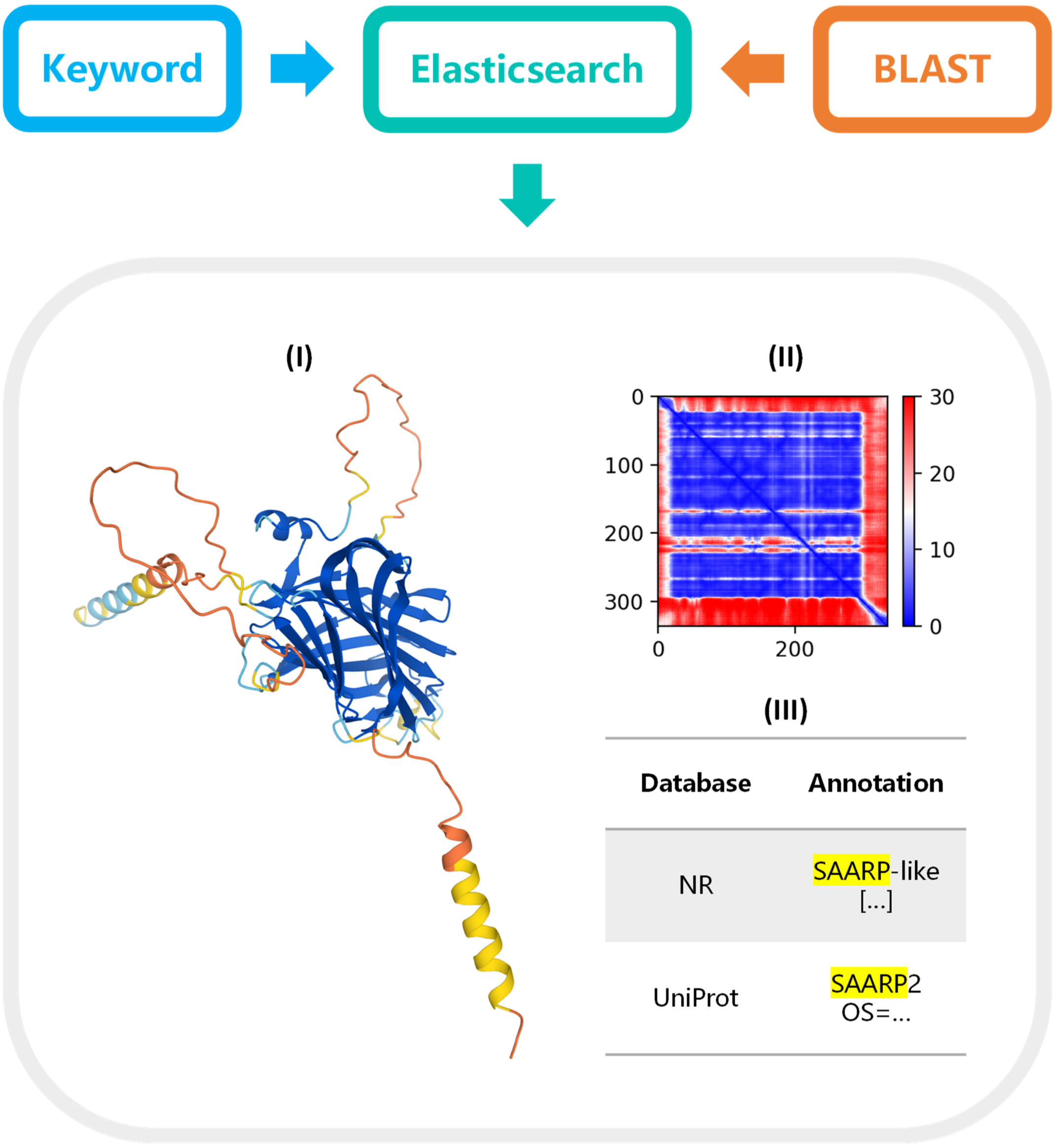

Fig S1-S4

Click here to access/download
Supplementary Material
SI.docx

Table S1

Click here to access/download
Supplementary Material
Table_S1.xlsx

Table S2

Click here to access/download
Supplementary Material
Table_S2.xlsx

Table S3

Click here to access/download
Supplementary Material
Table_S3.xlsx

Table S4

Click here to access/download
Supplementary Material
Table_S4.xlsx

Table S5

Click here to access/download
Supplementary Material
Table_S5.xlsx

Video S1

Click here to access/download
Supplementary Material
Video_S1.mp4
