# Supplementary material for: Utilizing an artificial intelligence system to build the digital structural proteome of reef-building corals
Source: Gigascience. 2022 Nov 18;11:giac117. doi: 10.1093/gigascience/giac117 (PMC9673494; doi:10.1093/gigascience/giac117)
Supplement: giac117_Supplemental_Files [file giac117_supplemental_files.zip › SI.docx]

**Supplemental information**

**Fig S1. Statistics of Pfam annotation.** Horizontal axis is protein number and vertical axis is Pfam family name. Top 50 Pfam families with largest counts are displayed.

**Fig S2. Venn plot of template domain distribution among ColabFold search results, SCOP and CATH.**

**Fig S3. Statistics of RMSDs between ColabFold and AlphaFold predictions.**

**
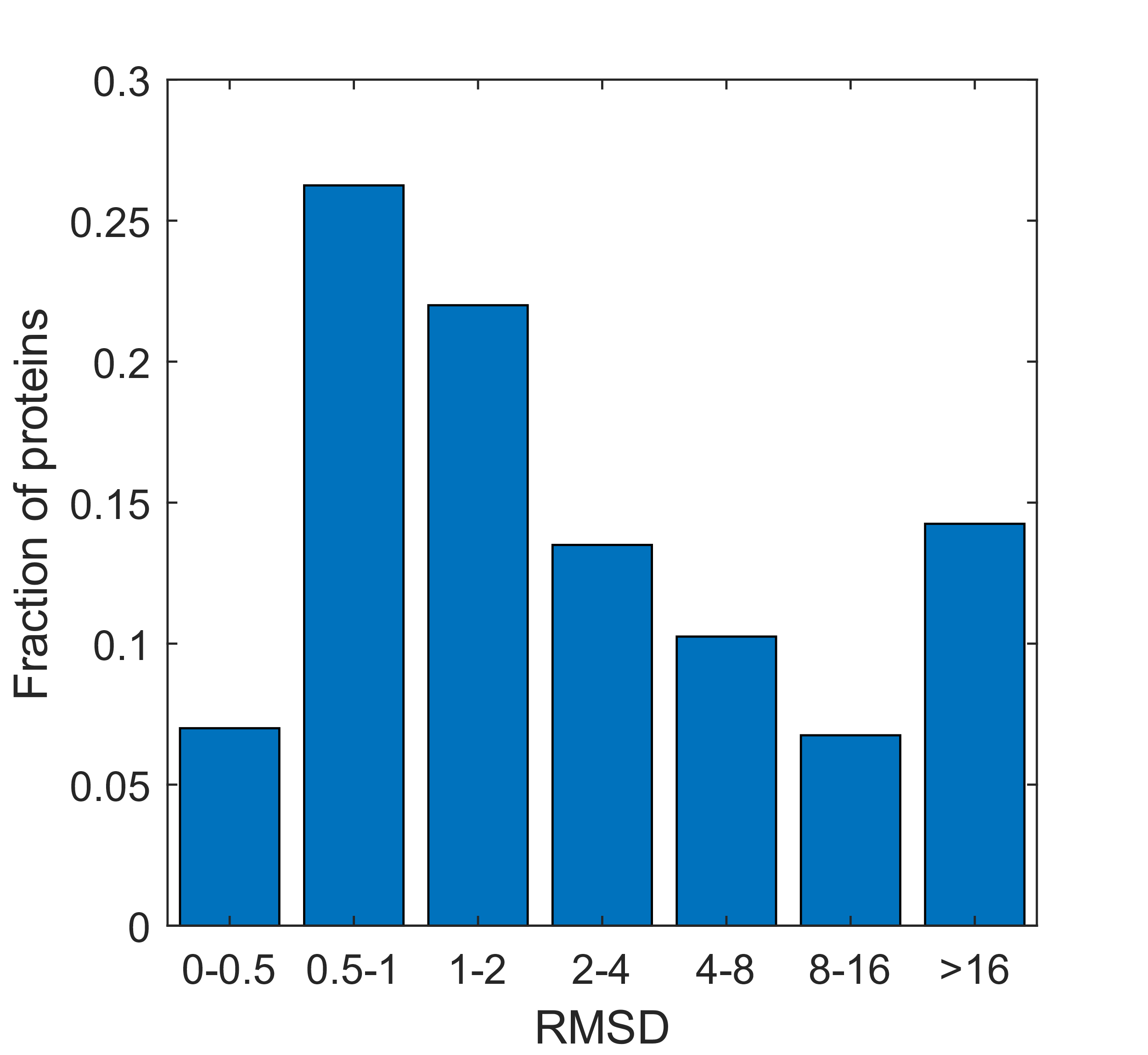
**

**Fig S4. Structures of USOMP-like proteins.** Regions shaped like β-barrels are marked with red boxes.

**Table S1.** **Peptide fraction separation liquid chromatography elution gradient.**

**Table S2.** **Liquid chromatography elution gradient.**

**Table S3.** **Sequences and expression profiles of identified proteins.**

**Table S4. Model scores, annotations and template domains of identified proteins.**

**Table S5. RMSDs between ColabFold and AlphaFold predictions.**

**Video S1. Demo of searching skeletal aspartic acid-rich protein via web interface of CP-8382.**
