# Supplementary material for: Utilizing an artificial intelligence system to build the digital structural proteome of reef-building corals
Source: Gigascience. 2022 Nov 18;11:giac117. doi: 10.1093/gigascience/giac117 (PMC9673494; doi:10.1093/gigascience/giac117)
Supplement: giac117_Response_to_Reviewer_Comments_Original_Submission [file giac117_response_to_reviewer_comments_original_submission.pdf]

Author's Response To Reviewer Comments

Close

Dear Professor Zhou,

Thank you very much for giving us an opportunity to revise our manuscript Utilizing artificial intelligence system to build the digital structural proteome of reef-building corals (GIGA-D-22-00178). We feel obliged to express our great appreciation to all the editors and reviewers for the valuable comments and suggestions. We have carefully revised and supplemented our manuscript according to each comment, and all corrections are marked with different colours.
The main corrections in the paper and the responses to the comments are as following:

Reviewer #1:
1. Response to comment: I would encourage the authors to spend more effort in analyzing the sequences.
Thank you for this important reminder. Although it might not be required for the data note category, bioinformatics analysis will undoubtedly enhance the value of the publication. We have supplemented Pfam annotations (new Fig S1) as well as more sequence analyses (new Fig 1).

2. Response to comment: It may be meaningful to compare the predicted structure models to the SCOP or CATH database to see the fold distribution and if there is any new fold.
We have made an attempt to compare the predicted structure models to SCOP and CATH. ColabFold totally found 27,887 template domains, among which nearly 70% have been registered in SCOP or CATH (new Fig S2). Nevertheless, current template search results generated by ColabFold or AlphaFold turn out to be sequence-based, not final-structure-based, thus using them to represent fold distributions might fail to avoid false positives. From our perspective, it would be better to apply high-throughput structure alignment methods into classification of remaining template domains as well as annotation of various novel folds, yet existing methods seem to be not efficient enough to handle the increasing scale of data pushed from AI systems such as ColabFold.

3. Response to comment: What happened to the ~200 proteins that ColabFold failed to work?
ColabFold on Nvidia Tesla V100 (32G GPU memory) cannot process proteins larger than ~2550 AA. The maximum size of structure predictions is determined by GPU memory currently, even AlphaFold Protein Structure Database can only split large proteins (longer than 2700AA) into 1400AA fragments for structure prediction (https://www.alphafold.ebi.ac.uk/download). Since predicting structures of long fragments of the remaining 216 proteins (933,369 residues, average length 4,321 AA) may be time-consuming while one of our authors need the publication of this paper for his PhD, we fail to add this work to our revised manuscript, but we will gradually add these partial structure predictions to our web server. Besides, we are negotiating with colleagues to borrow a Nvidia Tesla A100 (80G GPU memory) for directly structure prediction of some long proteins.

4. Response to comment: I suggest adding a browse function to the server for browsing the data.
Browse function has been added at http://corals.bmeonline.cn/prot/browse.php.

Reviewer #2:
1. Response to comment: Please ensure that the entire structure repository is available for unrestricted download as per http://corals.bmeonline.cn/prot/release.php; The link from 'CoralBioinfo' gives a 404 error: http://corals.bmeonline.cn/index.php; In http://corals.bmeonline.cn/blast/, please include a link back to http://corals.bmeonline.cn/prot/.
Thank you for the suggestions above. All requirements have been completed.

2. Response to comment: Please replace 'co-expression' with 'homologous proteins'. Use full genus names on first use. Proofreading of grammar required throughout, and elimination of non-scientific phrasing.
Thank you for this important reminder. All incorrect terms have been corrected.

3. Response to comment: Although the manuscript lacks bioinformatic analysis of the structural proteome, this is not required for the data note category but would enhance the value of the publication if provided.
We have supplemented more sequence analyses (new Fig 1, new Fig S1) and made an attempt to compare the predicted structure models to SCOP and CATH (new Fig S2).

4. Response to comment: Please report the RMSD between structures predicted in this work with the published alphafold structures for the same proteins.
From our experience, RMSD is usually calculated between different conformations of the same protein, while it is hard to find the same proteins (exactly same elements and same order) for our coral proteins in AlphaFold DB. By the aid of PyMOL, we selected 400 structures and aligned them to public AlphaFold structures of their similar proteins (BLAST E value < 2.8e-309) instead, then calculated RMSD. Most structure pairs seem to have little difference (Fig S3), but some do differ significantly. Considering the lack of exactly same coral proteins in AlphaFold DB, it might be difficult to detect whether the sequential feature or the AI system itself is responsible for those differences. It is proposed that until more AlphaFold-registered or experimentally verified structures are available, pLDDT will remain the main technical control indicator for coral protein structure prediction, meanwhile our resulting dataset may temporarily serve as a coral-specific extension of AlphaFold DB for RMSD analysis (coral biologists can search our dataset for the same proteins and calculate RMSD).

5. Response to comment: The data dump would be stronger if the mass spec proteomics data was also made available. What proportion of the structural proteome has mass-spectral support?
The mass spectrometry proteomics data have been deposited to the ProteomeXchange Consortium via the iProX partner repository with the dataset identifier PXD034973, and they will be made public once our manuscript is published. All the 8,382 proteins have mass-spectral support, and quantification of their expression level is available in Table S3.

6. Response to comment: Please include a supplementary text file containing the key features of each predicted protein.
We have provided Table S4 for all key features. We have also developed a browse page (http://corals.bmeonline.cn/prot/browse.php) for our web server.

7. Response to comment: The long proteins could be split by domain to provide some structural information.
This suggestion is excellent. For long proteins, AlphaFold Protein Structure Database just split them into 1400AA fragments with 200AA overlap. Splitting long proteins by domain before prediction should be more appropriate, yet there is no existing tool for this. Since predicting structures of long fragments of the remaining 216 proteins (933,369 residues, average length 4,321 AA) may be time-consuming while one of our authors need the publication of this paper for his PhD, we fail to add this work to our revised manuscript, but we will gradually add these partial structure predictions to our web server, and we will try to customize a pipeline following your suggestions and apply it into subsequent studies. Besides, we are negotiating with colleagues to borrow a Nvidia Tesla A100 (80G GPU memory) for directly structure prediction of some long proteins.

8. Response to comment: To boost the value of this data, the authors might also consider predicting the coral symbiont proteomes followed by integrative analysis of host and symbiont proteomes to predict interacting partners.
This work has been already planned. We have generated the multiple sequence alignments of more than 90,000 Symbiont proteins for structure prediction, and the Big Data Computing Center of Southeast University have promised to triple the number of available GPUs for us if our manuscript is accepted. Of course, we will prioritize the remaining 216 long proteins in this work once we receive added computing resources.

9. Response to comment: What are the domain and sequence features of the low and very-low confidence predictions?
They are “orphan sequences” with few homologous sequences. AI systems depending on coevolutionary information do not do well with these sequences.

10. Response to comment: Is the reference genome available for any species? How does the mass spec and structural data improve the genome annotation and vice versa?
Our team has assembled the genomes of investigated corals to the chromosome level, yet whole genome annotation is still a work in progress. From our experience, mass spectrometry technology

enables researchers to obtain high-throughput protein information from relatively small samples in standardized steps, and structural data enable researchers to use computational biology methods (such as molecular docking, https://doi.org/10.3389/fphys.2021.759370) to find key proteins and explore crucial physiological functions, providing evidence more reliable than sequence alignment. It is particularly beneficial for studies on several non-model organisms represented by corals. These species may be important to our ecology and society, but existing experimental protocols for them are not as perfect as those for model organisms, moreover people tend to focus on their potentially valuable components rather than a head-to-tail understanding of them.

11. Response to comment: At present large parts of the discussion are irrelevant.
We apologize for our first author's overexcitement at the time. We have deleted irrelevant arguments regarding COVID 19 and the call to arms for bioinformaticians.

12. Response to comment: Comment on whether toxicity is reported for these coral species.
No comment on toxicity is reported for these coral species, yet several toxic substances were found in their relatives (Acropora digitifera, Gacesa R, 2015; Montipora capitata, Hagedorn M, 2015). From our experience, our investigated corals are unlikely to be as dangerous as Millepora corals.

We look forward to hearing from you regarding our submission, and we would be glad to respond to any further questions and comments that you may have.

Yours sincerely, on behalf of all the authors,

Chunpeng He
State Key Laboratory of Bioelectronics, School of Biological Science and Medical Engineering, Southeast University, Nanjing, China.
e-mail: cphe@seu.edu.cn

Zuhong Lu
State Key Laboratory of Bioelectronics, School of Biological Science and Medical Engineering, Southeast University, Nanjing, China.
e-mail: zhlu@seu.edu.cn

Close
