# Supplementary material for: Utilizing an artificial intelligence system to build the digital structural proteome of reef-building corals
Source: Gigascience. 2022 Nov 18;11:giac117. doi: 10.1093/gigascience/giac117 (PMC9673494; doi:10.1093/gigascience/giac117)
Supplement: giac117_Response_to_Reviewer_Comments_Revision_1 [file giac117_response_to_reviewer_comments_revision_1.pdf]

Author's Response To Reviewer Comments

Close

Dear Professor Zhou,

Thank you very much for your approval and advice for our manuscript Utilizing artificial intelligence system to build the digital structural proteome of reef-building corals (GIGA-D-22-00178). We have carefully revised and supplemented our manuscript according to each comment, and the main corrections in the paper and the responses to the comments are as following:

Reviewer #1:
Thank you very much for your approval !

Reviewer #2:
1. Response to comment: Careful English language revision is required.
We have polished our manuscript again. Sorry for the hurry when we wrote this manuscript. The academic pressure might make our first author too nervous and hasty.

2. Response to comment: My understanding from the original text was that the 8382 'co-expressed' proteins were homologues.
We are afraid that your previous understanding was right. The 8382 proteins are all 3-way homologues among our corals, for we didn't have extra computing resources for annotation or structure prediction of any other protein at that time. We have supplemented necessary details to avoid misunderstandings, and please allow us to keep the current statements instead of drawing a new Venn diagram with six zeros.

3. Response to comment: Table S4 please make this a clean excel table.
Thank you for this important reminder. We have formatted the main sheet of it and moved all JSON-form text to another sheets. The previous JSON format is programming-friendly rather than reader-friendly.

We look forward to hearing from you regarding our submission, and we would be glad to respond to any further questions and comments that you may have.

Yours sincerely, on behalf of all the authors,

Chunpeng He
State Key Laboratory of Bioelectronics, School of Biological Science and Medical Engineering, Southeast University, Nanjing, China.
e-mail: cphe@seu.edu.cn

Zuhong Lu
State Key Laboratory of Bioelectronics, School of Biological Science and Medical Engineering, Southeast University, Nanjing, China.
e-mail: zhlu@seu.edu.cn

Close
