# Supplementary material for: Utilizing an artificial intelligence system to build the digital structural proteome of reef-building corals
Source: Gigascience. 2022 Nov 18;11:giac117. doi: 10.1093/gigascience/giac117 (PMC9673494; doi:10.1093/gigascience/giac117)
Supplement: giac117_Response_to_Reviewer_Comments_Revision_2 [file giac117_response_to_reviewer_comments_revision_2.pdf]

Author's Response To Reviewer Comments

Close

Dear Professor Zhou,

Thank you very much for your approval and advice for our manuscript Utilizing artificial intelligence system to build the digital structural proteome of reef-building corals (GIGA-D-22-00178). We have highlighted in _R2 that all 8382 proteins are 3-way homologues (protein A in coral species A has high homologs in both species B and C), not just a mix of proteins from the three coral species (total protein number of three corals should be 60,000~75,000). It's not rare for scleractinians to have so many highly similar genes even if their species diversity is extremely high (Shinzato C, 2021). If statistics were separated by species, there would be just three identical subgraphs with three different species names. Reviewer 2 might think there were several 2-way homologues in our dataset, thus he gave us option 1 in comments on the _R1 review to draw a Venn graph for distinguishment. But given that all proteins are 3-way homologues, all of them will locate in the center of Venn plot, making it not quite meaningful. Thus, please allow us to keep the current figures and tables, for we are afraid that splitting them will make our manuscript sort of repetitive rather than clear.
We don't see any further comments from reviewer 2 yet he seems to ask for minor revision again. Are there any bugs, or you have included his comments in the author decision letter?

We look forward to hearing from you regarding our revision, and we hope our coral protein structure dataset will receive the attention as soon as possible.

Yours sincerely, on behalf of all the authors,

Chunpeng He
State Key Laboratory of Bioelectronics, School of Biological Science and Medical Engineering, Southeast University, Nanjing, China.
e-mail: cphe@seu.edu.cn

Zuhong Lu
State Key Laboratory of Bioelectronics, School of Biological Science and Medical Engineering, Southeast University, Nanjing, China.
e-mail: zhlu@seu.edu.cn

Close
