# Supplementary material for: Utilizing an artificial intelligence system to build the digital structural proteome of reef-building corals
Source: Gigascience. 2022 Nov 18;11:giac117. doi: 10.1093/gigascience/giac117 (PMC9673494; doi:10.1093/gigascience/giac117)
Supplement: giac117_Reviewer_1_Report_Original_Submission — Jianyi Yang -- 8/31/2022 Reviewed [file giac117_reviewer_1_report_original_submission.pdf]

Reviewer Report

Title: Utilizing artificial intelligence system to build the digital structural proteome of reef-building corals

Version: Original Submission **Date:** 8/31/2022

Reviewer name: Jianyi Yang

Reviewer Comments to Author:

The authors present the predicted structures for the proteome of the reef-building corals. 8382 protein sequences were obtained by experiments, which are fed into ColabFold for structure modeling, generating 8166 structure models. Overall, this is a valuable study toward the understanding of the reef-building coral. Here are a few comments for possible improvement.
1. It becomes trivial for proteome-wide structure predictions nowadays with AlphaFold2 and other methods. I think the major contribution of the current study is the determination of the proteome sequences rather than the structure prediction. Thus, I would encourage the authors to spend more effort in analyzing the sequences, for example, how the sequences cover the Pfam families, how redundant the sequences are, how much they overlap with the sequences in UniProt, etc.
2. It may be meaningful to compare the predicted structure models to the SCOP or CATH database to see the fold distribution and if there is any new fold.
3. What happened to the ~200 proteins that ColabFold failed to work?
4. I suggest adding a browse function to the server for browsing the data.

Level of Interest

Please indicate how interesting you found the manuscript: Choose an item.

Quality of Written English

Please indicate the quality of language in the manuscript: Choose an item.

Declaration of Competing Interests

Please complete a declaration of competing interests, considering the following questions:

- Have you in the past five years received reimbursements, fees, funding, or salary from an organisation that may in any way gain or lose financially from the publication of this manuscript, either now or in the future?
- Do you hold any stocks or shares in an organisation that may in any way gain or lose financially from the publication of this manuscript, either now or in the future?

- Do you hold or are you currently applying for any patents relating to the content of the manuscript?
- Have you received reimbursements, fees, funding, or salary from an organization that holds or has applied for patents relating to the content of the manuscript?
- Do you have any other financial competing interests?
- Do you have any non-financial competing interests in relation to this paper?

If you can answer no to all of the above, write 'I declare that I have no competing interests' below. If your reply is yes to any, please give details below.

I declare that I have no competing interests.

I agree to the open peer review policy of the journal. I understand that my name will be included on my report to the authors and, if the manuscript is accepted for publication, my named report including any attachments I upload will be posted on the website along with the authors' responses. I agree for my report to be made available under an Open Access Creative Commons CC-BY license (http://creativecommons.org/licenses/by/4.0/). I understand that any comments which I do not wish to be included in my named report can be included as confidential comments to the editors, which will not be published.

Choose an item.

To further support our reviewers, we have joined with Publons, where you can gain additional credit to further highlight your hard work (see: https://publons.com/journal/530/gigascience). On publication of this paper, your review will be automatically added to Publons, you can then choose whether or not to claim your Publons credit. I understand this statement.

Yes Choose an item.
