# Supplementary material for: Utilizing an artificial intelligence system to build the digital structural proteome of reef-building corals
Source: Gigascience. 2022 Nov 18;11:giac117. doi: 10.1093/gigascience/giac117 (PMC9673494; doi:10.1093/gigascience/giac117)
Supplement: giac117_Reviewer_2_Report_Original_Submission — Brendan Robert E. Ansell, PhD -- 9/26/2022 Reviewed [file giac117_reviewer_2_report_original_submission.pdf]

Reviewer Report

Title: Utilizing artificial intelligence system to build the digital structural proteome of reef-building corals

Version: Original Submission **Date:** 9/26/2022

Reviewer name: Brendan Robert E. Ansell, PhD

Reviewer Comments to Author:

Zhu and colleagues report the generation of predicted protein structures via alpha-fold, for three coral species: A muricate, M foliosa and P verrucosa. Mass-spec analysis of the proteome of the three species is also performed. The authors describe a handful of structures that appear to be orthologues across the species and may have functions as pore-forming toxins, in calcium deposition and host-symbiont interactions.

The generated protein structures will be of use to the scientific community and the web server is quite good.

Major comments:

Please ensure that the entire structure repository is available for unrestricted download as per http://corals.bmeonline.cn/prot/release.php

Incorrect use of 'co-expression'. Assume the authors mean protein orthologues (i.e., homologues across species). Please replace with 'homologous proteins' throughout including in http://corals.bmeonline.cn/prot/release.php

The link from 'CoralBioinfo' gives a 404 error: http://corals.bmeonline.cn/index.php

In http://corals.bmeonline.cn/blast/, please include a link back to http://corals.bmeonline.cn/prot/

Although the manuscript lacks bioinformatic analysis of the structural proteome, this is not required for the data note category but would enhance the value of the publication if provided.

In terms of validation, there is a technical control for the alphafold instance that this project used, which the authors should include. Specifically, please report the RMSD between structures predicted in this work with the published alphafold structures for the same proteins Acropora muricata (20 proteins), Montipora foliosa (8 proteins) and Pocillopora verrucosa (70 proteins), available at e.g. https://alphafold.ebi.ac.uk/search/text/Montipora%20foliosa%20?organismScientificName=Montipora%20foliosa

Please detail in methods how the mass spec data relates to improving the genome or proteome annotation of each species. How was the mass spec data used? I presume it was used to identify 3-way orthologues between the species, and producing the "8,382 co-expressed proteins" that were selected for structural prediction. The data dump would be stronger if the mass spec proteomics data was also made available. What proportion of the structural proteome has mass-spectral support?

Please include a supplementary text file containing the key features of each predicted protein e.g. % high confidence structure, gene id, interpro domain annotations , and top blast homologues.

The long proteins could be split by domain to provide some structural information.

To boost the value of this data, the authors might also consider predicting the coral symbiont proteomes

followed by integrative analysis of host and symbiont proteomes to predict interacting partners.
What are the domain and sequence features of the low and very-low confidence predictions?
Is the reference genome available for any species? What is the completeness and content. How does the mass spec and structural data improve the genome annotation and vice versa?
At present large parts of the discussion are irrelevant. Comments about covid-19 and the role of bioinformaticians are outside the scope of a research report.
Minor comments:
Comment on whether toxicity is reported for these coral species.
Use full genus names on first use
Proofreading of grammar required throughout, and elimination of non-scientific phrasing. Drop irrelevant arguments regarding COVID 19 and the call to arms for bioinformaticians.

Level of Interest

Please indicate how interesting you found the manuscript: Choose an item.

Quality of Written English

Please indicate the quality of language in the manuscript: Choose an item.

Declaration of Competing Interests

Please complete a declaration of competing interests, considering the following questions:

- Have you in the past five years received reimbursements, fees, funding, or salary from an organisation that may in any way gain or lose financially from the publication of this manuscript, either now or in the future?
- Do you hold any stocks or shares in an organisation that may in any way gain or lose financially from the publication of this manuscript, either now or in the future?
- Do you hold or are you currently applying for any patents relating to the content of the manuscript?
- Have you received reimbursements, fees, funding, or salary from an organization that holds or has applied for patents relating to the content of the manuscript?
- Do you have any other financial competing interests?
- Do you have any non-financial competing interests in relation to this paper?

If you can answer no to all of the above, write 'I declare that I have no competing interests' below. If your reply is yes to any, please give details below.

I declare that I have no competing interests'

I agree to the open peer review policy of the journal. I understand that my name will be included on my report to the authors and, if the manuscript is accepted for publication, my named report including any attachments I upload will be posted on the website along with the authors' responses. I agree for my report to be made available under an Open Access Creative Commons CC-BY license (http://creativecommons.org/licenses/by/4.0/). I understand that any comments which I do not wish to be included in my named report can be included as confidential comments to the editors, which will not be published.

Choose an item.

To further support our reviewers, we have joined with Publons, where you can gain additional credit to further highlight your hard work (see: https://publons.com/journal/530/gigascience). On publication of this paper, your review will be automatically added to Publons, you can then choose whether or not to claim your Publons credit. I understand this statement.

Yes Choose an item.
