# Supplementary material for: Utilizing an artificial intelligence system to build the digital structural proteome of reef-building corals
Source: Gigascience. 2022 Nov 18;11:giac117. doi: 10.1093/gigascience/giac117 (PMC9673494; doi:10.1093/gigascience/giac117)
Supplement: giac117_Reviewer_2_Report_Revision_1 — Brendan Robert E. Ansell, PhD -- 10/5/2022 Reviewed [file giac117_reviewer_2_report_revision_1.pdf]

Reviewer Report

Title: Utilizing artificial intelligence system to build the digital structural proteome of reef-building corals

Version: Revision 1 **Date:** 10/5/2022

Reviewer name: Brendan Robert E. Ansell, PhD

Reviewer Comments to Author:

I appreciate the authors' efforts to address my comments.
The manuscript requires further revisions before it is of suitable quality for publication in Gigascience.
Careful English language revision is required.
My understanding from the original text was that the 8382 'co-expressed' proteins were homologues.
For example, protein A in coral species A, should have a homolog in either species B, or species C (or both- making it a 3-way homolog).
From Figure 1a, it appears that the 8k proteins are just a mix of proteins from the three coral species.
This does not make them homologues.
I think the confusion comes from searching spectra against PRJNA544778, UniProt and NR databases.
This step allows you to identify 'BLAST homologues', and to annotate the peptide. The homology I'm interested in is between the three species, rather than between the query peptide and reference database. â€¨
So my original comment (# 2) about replacing 'co-expressed' with 'homologue' doesn't apply. Neither term is appropriate.
I think at this stage the authors have two options:
1, if the proteins are homologous to each other, please include a Venn diagram or Upset plot to display the homologues between the three coral species (.
2, if the proteins are a mix of whatever could be sequenced on mass spec, then do not refer to them as 'homologs', and simply include the number of proteins sequenced from each species.
In the case of option 2, please break up (facet, or fill colour) Figure 1c bars by species. The same applies for supplementary figures e.g. Fig S3 - please split by coral species. This allows the reader to see whether the structure prediction quality is higher for some species than others.
Table S4 please make this a clean excel table. Separate NR fields by ":", remove the quotes and curly braces etc. Please make it easy for the research community to use this table.

Level of Interest

Please indicate how interesting you found the manuscript: Choose an item.

Quality of Written English

Please indicate the quality of language in the manuscript: Choose an item.

Declaration of Competing Interests

Please complete a declaration of competing interests, considering the following questions:

- Have you in the past five years received reimbursements, fees, funding, or salary from an organisation that may in any way gain or lose financially from the publication of this manuscript, either now or in the future?
- Do you hold any stocks or shares in an organisation that may in any way gain or lose financially from the publication of this manuscript, either now or in the future?
- Do you hold or are you currently applying for any patents relating to the content of the manuscript?
- Have you received reimbursements, fees, funding, or salary from an organization that holds or has applied for patents relating to the content of the manuscript?
- Do you have any other financial competing interests?
- Do you have any non-financial competing interests in relation to this paper?

If you can answer no to all of the above, write 'I declare that I have no competing interests' below. If your reply is yes to any, please give details below.

I declare that I have no competing interests

I agree to the open peer review policy of the journal. I understand that my name will be included on my report to the authors and, if the manuscript is accepted for publication, my named report including any attachments I upload will be posted on the website along with the authors' responses. I agree for my report to be made available under an Open Access Creative Commons CC-BY license (http://creativecommons.org/licenses/by/4.0/). I understand that any comments which I do not wish to be included in my named report can be included as confidential comments to the editors, which will not be published.

Choose an item.

To further support our reviewers, we have joined with Publons, where you can gain additional credit to further highlight your hard work (see: https://publons.com/journal/530/gigascience). On publication of this paper, your review will be automatically added to Publons, you can then choose whether or not to claim your Publons credit. I understand this statement.

Yes Choose an item.
